# Highly Selective and Scalable Molecular Fluoride Sensor for Naked-Eye Detection

**DOI:** 10.1021/acsami.4c01187

**Published:** 2024-03-30

**Authors:** Zakir Ullah, Saravanan Subramanian, Haeseong Lim, Nesibe A. Dogan, Joo Sung Lee, Thien S. Nguyen, Cafer T. Yavuz

**Affiliations:** ‡ Institut de Ciència de Materials de Barcelona (ICMAB), Consejo Superior de Investigaciones Científicas (CSIC), Campus Universitari de Bellaterra, Cerdanyola del Vallès 08193, Spain; § Inorganic Materials and Catalysis Division, Council of Scientific and Industrial Research (CSIR)−Central Salt & Marine Chemicals Research Institute, Bhavnagar, Gujarat 364002, India; ∥ Department of Materials Science and Engineering, Korea Advanced Institute of Science & Technology (KAIST), 291 Daehak-ro, Yuseong-gu, Daejeon 34141, Republic of Korea; ⊥ Department of Chemical and Biomolecular Engineering, Korea Advanced Institute of Science & Technology (KAIST), Daejeon 34141, Republic of Korea; # Oxide and Organic Nanomaterials for Energy and Environment (ONE) Lab, Chemistry Program, Advanced Membranes & Porous Materials Center, KAUST Catalysis Center, Physical Science & Engineering (PSE), King Abdullah University of Science and Technology (KAUST), Thuwal 23955, Saudi Arabia

**Keywords:** water treatment, point-of-use care, fluoride contamination, hydrogen-bonded interaction, scale up

## Abstract

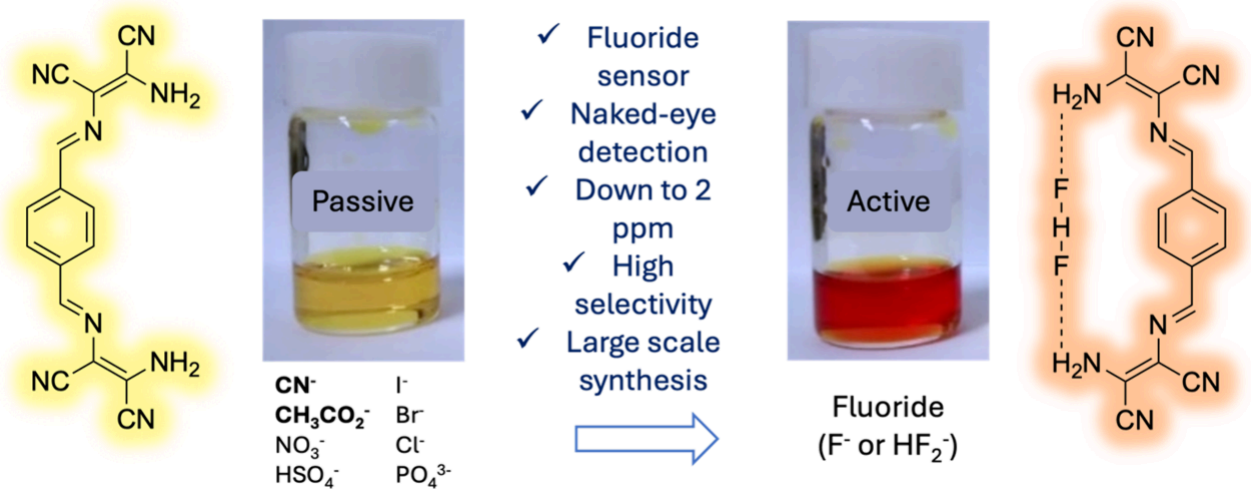

Fluoride is widely present in nature, and human exposure to it is generally regarded as inevitable. High levels of fluoride intake induce acute and chronic illnesses. To reduce potential harm to the general public, it is essential to create selective fluoride detectors capable of providing a colorimetric response for naked-eye detection without the need for sophisticated equipment. Here, we report a one-pot synthesis of four different diaminomaleonitrile-derived Schiff base sensors. The terephthalaldehyde adduct provided a strong color change visible to the naked eye at a F^–^ concentration level as low as 2 ppm. From the evaluation against other anions, such as CN^–^, I^–^, Br^–^, Cl^–^, NO_3_^–^, PO_4_^3–^, OAc^–^, and HSO_4_^–^, the molecular sensor displayed a visible color change exclusively upon exposure to fluoride, underscoring exceptional selectivity. As a key intermediate for understanding the mechanism, HF_2_^–^ was confirmed by ^19^F nuclear magnetic resonance. Theoretical calculations suggested a deprotonation-triggered bathochromic shift brought about by the unique electronic structure of the sensor. Furthermore, the simple synthetic protocol from economically accessible materials allowed for the preparation of the compound on a large scale, rendering it a highly practical visual fluoride sensor.

## Introduction

The quality of drinking water is a major concern in daily life and will continue to become more important as water streams are increasingly threatened by conventional and emerging contaminants.^1−3^ Fluoride is both a nutrient and a toxin depending upon its amount.

Accumulation of fluoride from drinking water has detrimental effects on human health. Conditions such as osteoporosis, dental or skeletal fluorosis, metabolic dysfunctions, and kidney problems have been documented to occur as a result of fluoride overexposure.^4−6^ The United States Environmental Protection Agency (U.S. EPA) hence has issued enforceable and non-enforceable limits of the fluoride level in drinking water sources at 4 and 2 ppm, respectively.^7^ It is, therefore, of great importance to develop an easy-to-use, highly sensitive fluoride detection method to prevent detrimental impacts on human health.^8,9^

Fluoride, with its significant roles in chemical, biological, environmental, and industrial processes,^10^ has the capacity to create strong hydrogen bonds as a result of being the anion with the highest charge density. The main objective of an astute fluoride sensor can be divided into three major factors: response time, sensitivity at a low concentration, and user-friendly data presentation. Detection of fluoride ions can involve various mechanisms, such as hydrogen bonding,^11^ Lewis acid coordination, and the generation of new species following the reaction with F^–^. In terms of sensing media, organic solvents, such as dimethyl sulfoxide (DMSO),^12−14^ chloroform,^15,16^ acetonitrile,^17^ tetrahydrofuran (THF),^18^ and solvent mixtures,^19,20^ are favored in many cases. This is because the high hydration enthalpy in water leads to reduced binding efficiencies. It is crucial to note that the majority of reported detectors have showcased their sensing capabilities using tetrabutylammonium fluoride salt (TBAF) rather than inorganic fluoride salts as a result of the higher solubility of the former in organic solvents. Various approaches aimed at water detection have been reported as well. Some of these methods involve the use of inorganic NaF salt^21^ with heightened detection performance by increasing salt concentrations^22^ or employing specific pH buffers,^23^ surfactants,^6^ and co-solvents^24^ to enhance solubility. Additional strategies used to trigger the sensing process include the use of metal ions, the incorporation of sensor molecules within macrocycles,^25^ and the employment of container-like structures.^26^

The presence of competing anions, e.g., cyanide and acetate, impose a requirement of highly sensitive F^–^ sensors to have accurate qualitative and quantitative assessments of the fluoride content.^18^ A number of selective fluoride detection methods are available, such as ion-selective electrodes, nuclear magnetic resonance (NMR), colorimetric analysis [ultraviolet (UV)], fluorescence sensing, and mass spectrometry. However, most of these methods require trained personnel, complex equipment, and laboratory setup. Considering the ease of operation, visual detection without the need for specialized equipment combined with a rapid response and high selectivity given by colorimetric sensors continues to be the favored choice. Herein, we report a highly selective molecular fluoride sensor featuring Schiff bases built upon commercially available 2,3-diaminomaleonitrile (DAMN) and terephthaladehyde. A systematic investigation of analogous structures revealed how factors such as resonance, the quantity of DAMN units, and the locations of substituents on the aromatic ring affected the colorimetric outcome. The detection limit was established at 2 ppm, aligning with the regulations set by the U.S. EPA. In addition, the simplicity of the structure and the ease of the synthesis enabled acquisition of the sensor in a large quantity, making it a highly accessible fluoride detector.

## Materials and Methods

### Materials

All solvents and reagents are purchased from Sigma-Aldrich and used as received without further purification.

### Sensor Synthesis and Characterization

All of the structures were synthesized by a Schiff base procedure. Aldehyde (10 mmol) reactant was dissolved in MeOH (50 mL) and stirred at room temperature. Diaminomaleonitrile was added to the solution (1 equiv for compounds **1** and **2** and 2 equiv for compounds **3** and **4**). Then, 3 drops of sulfuric acid were added to the mixture, and the mixture was stirred at room temperature for 6 h. The completion of the reaction was monitored, and subsequently, the precipitate was collected, washed with methanol, and dried at 80 °C. ^1^H and ^19^F NMR spectra were obtained from a Bruker NMR spectrometer. Absorption spectra were recorded using a Perkin-Elmer Lambda 1050 UV/vis spectrometer at room temperature. Fourier transform infrared (FTIR) spectra were measured by a Perkin-Elmer FTIR spectrometer. Large-scale synthesis of compound **4** was performed at a 100 mmol scale, and 30.2 g of the product was obtained (96%).

### Ultraviolet–Visible (UV–Vis) Measurements

#### Preparation of the Sensor Solution

A total of 0.1 mg of compound **4** was dissolved in 100 mL of THF (3.18 μM) (the solution of compound **4** gave intense signals that it had to be diluted 10 times compared to other structures to achieve the clear peak in the UV/vis spectrum). A total of 1 mg of other structures (compounds **1**, **2**, and **3**) was dissolved in THF (100 mL).

#### Selectivity Study for Anion Salts

A selectivity study for various anion salts was carried out using TBAF, TBAI, TBABr, TBACl, (CH_3_)_3_SiCN, KNO_3_, K_3_PO_4_, CH_3_COONa, and Na_2_SO_4_ as the anion sources. Each of the salts was dissolved in THF at 2 mM concentration. The solutions (1.5 mL) were introduced to a cuvette, and compound **4** solution (1.5 mL) was added. The UV/vis spectrum was obtained from the mixtures in cuvettes.

#### Detection Limit Study

TBAF (1 mL) was mixed with THF (99 mL) to make a 2614.6 ppm solution. A total of 10 mL of this solution was diluted with THF (90 mL) to make a 261.46 ppm solution. A total of 38.31 mL of the 261.46 ppm solution was diluted again with 61.69 mL of THF to make a 100 ppm solution. In this way, 100, 50, 25, 20, 10, 5, 2, 1, and 0.5 ppm levels of fluoride ion solution were obtained. After the fluoride ion solutions were obtained, compound **4** solution (1.5 mL) was mixed with each fluoride ion solution (1.5 mL) in cuvettes. The limit of detection was observed with each of the cuvettes on the UV/vis spectrometer in the range of 300–700 nm.

## Results and Discussion

### Sensor Synthesis and Characterization

**Figure 1:**
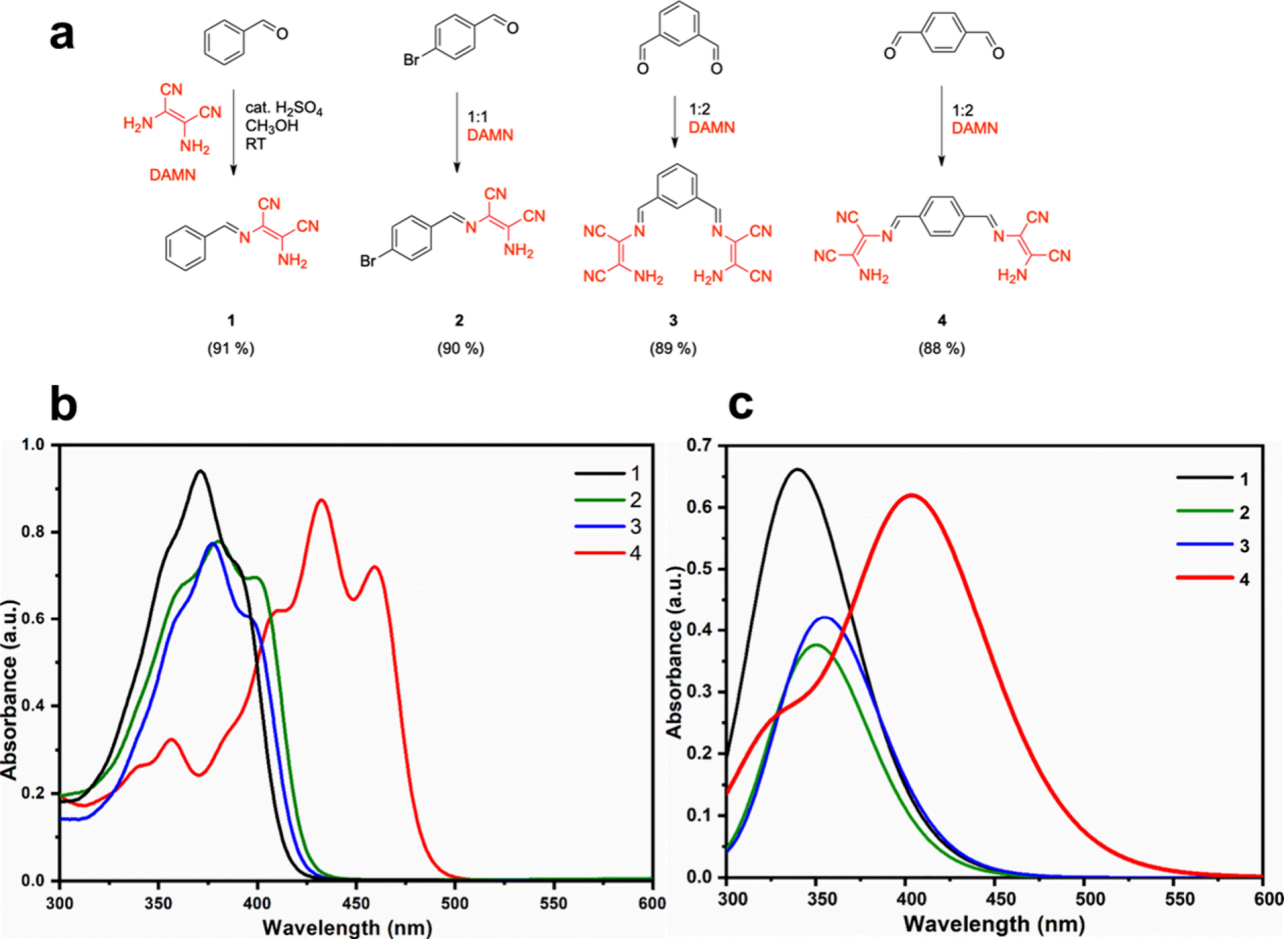
(a) Synthesis of structures of compounds **1**–**4**. (b) Experimental and (c) theoretical UV–vis spectra of compounds **1**–**4**.

**Figure 2:**
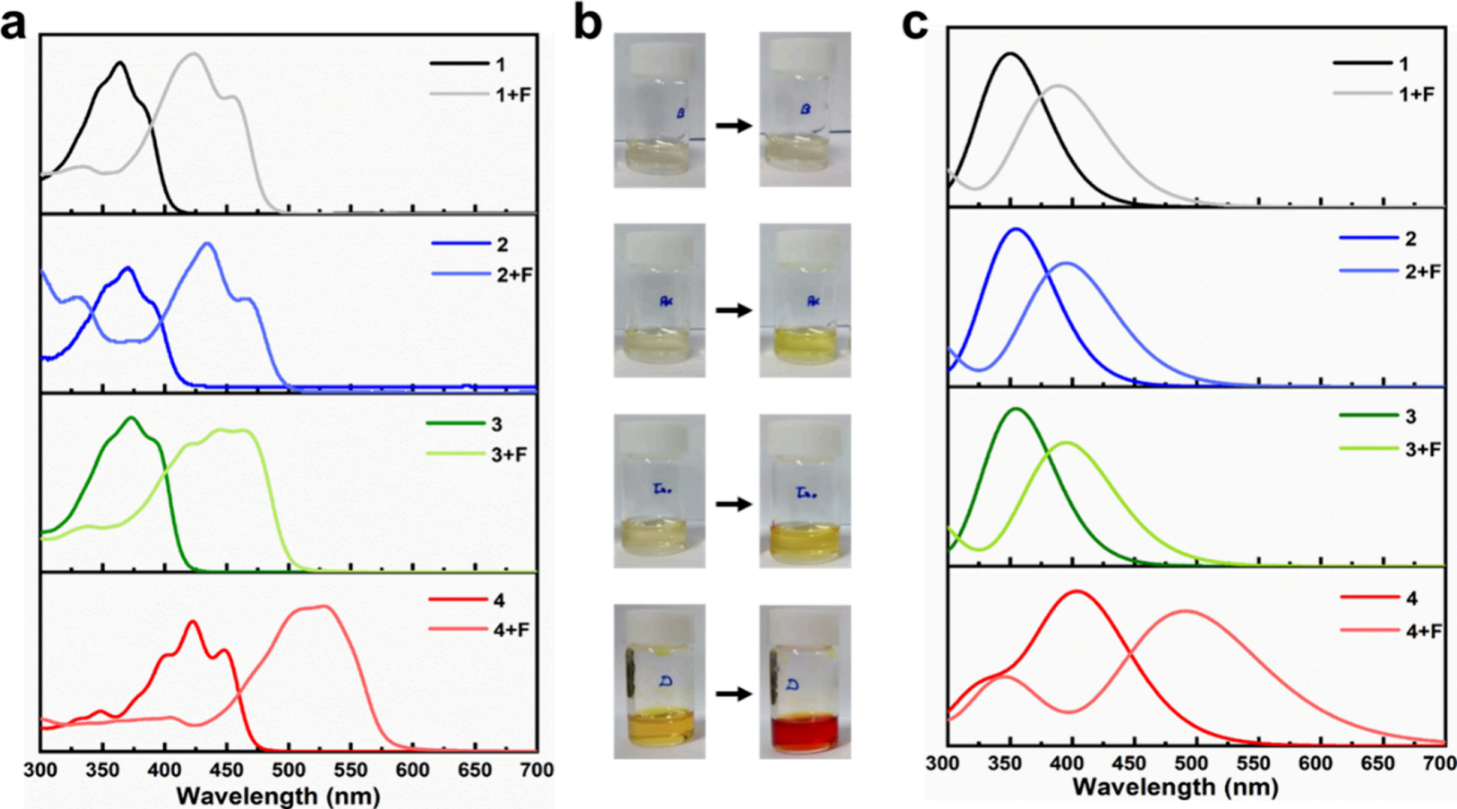
(a) Experimental UV–vis spectra of compounds **1** + F, **2** + F, **3** + F, and **4** + F. (b) Naked-eye detection and (c) theoretical UV–vis spectra of compounds **1** + F, **2** + F, **3** + F, and **4** + F. The UV–vis spectra were calculated at the CAM-B3LYP-D3/6-311G++(d,p)-CPCM level (solvent = THF).

The molecular sensors were synthesized using a conventional Schiff base methodology (Figure 1a).^19^ Compound **1** served as a negative control, in that no visible color change could be observed upon contact with fluoride. Compound **2** possessed an electron-withdrawing group (Br) in the *para* position of the benzene ring to show the effect of a withdrawing group, which was postulated to facilitate the mobility of the proton on the amino group, of which deprotonation would bring about extended π conjugation. In fact, while compound **2** showed a similar absorption spectrum to that of compound **1** before the introduction of F^–^, it resulted in a more pronounced color change after the addition of F^–^ (Figure 2). To further investigate the effect of DAMN, compounds **3** and **4** were designed with two DAMN units. The structures differed from the relative placement of DAMN units being *meta* and *para* in compounds **3** and **4**, respectively. Large-scale synthesis was also successfully achieved in the preparation of compound **4** with an even higher yield (96%), affording ∼30 g of product. All structures were characterized by ^1^H NMR and FTIR (Figures S1–S4 and S6–S9 of the Supporting Information) spectroscopy.

### Fluoride Detection

The experimental and simulated UV–vis spectra for compounds **1**–**4** are presented in panels b and c of Figure 1. The simulated spectra were in good agreement with the experimental data. The absorbance maxima for compounds **1**, **2**, and **3** appeared approximately at 350 nm, with compound **4** at 425 nm, following the same pattern as the experimental spectra. Such variation in absorption within the visible range made the impact of the placement of DAMN moieties in the structures evident. In particular, the extended conjugation system in compound **4** enabled by the *para* substitution pattern, depicted as the delocalization of the nitrogen lone pair on the amine across the π system to nitrile nitrogen on the other end of the molecule, brought about a red shift. Such continuous movement of the π electrons could not be obtained with the *meta* arrangement in compound **3**. Moreover, the proximity of the substituents in compound **3** might hinder the required planarity for effective conjugation because of a steric clash. In that sense, it is rational to observe that compounds **1**, **2**, and **3** exhibited similar spectra owing to the shorter conjugation.

Upon addition of F^–^, compound **1** showed a negligible color change, while compounds **2** and **3** displayed merely a slight color alteration from pale to dark yellow (Figure 2). In contrast, when TBAF was introduced into a solution of compound **4**, a pronounced and conspicuous transition in visual color occurred, shifting from dark yellow to deep red. The color could endure for at least 24 h unless acid was introduced to disrupt it. It is also worth noting that the concentration of compound **4** was one-tenth of that of the other structures. These colorimetric behaviors were spectroscopically confirmed by UV–vis spectra (Figure 2a). As shown, compounds **1**, **2**, and **3** showed a wavelength change from 350 to around 430 nm upon the addition of F^–^. This change would not produce a contrasting visual color shift when the complementary colors of these wavelengths are perceived by human eyes as yellow, only with different contrast. In the case of compound **4**, on the other hand, the transition was from 425 to around 520 nm. This resulted in a more distinct transformation of complementary colors, from yellow to red. Mechanistically, this intense visual bathochromic shift could presumably result from the further elongation of the π conjugation system, inferring a deprotonation of the amine proton. It is important to point out that the peak at 350 nm in the theoretical UV–vis spectrum of compound **4** + F (Figure 2c) was not observed in the experimental spectrum (Figure 2a). This absorption could presumably be assigned to the π–π* transition, calculated to be present by taking into account a complete deprotonation incidence. Therefore, the absence of this signal in the real spectrum implied partial or reversible proton abstraction by fluoride.

### Fluoride Sensing Selectivity

**Figure 3:**
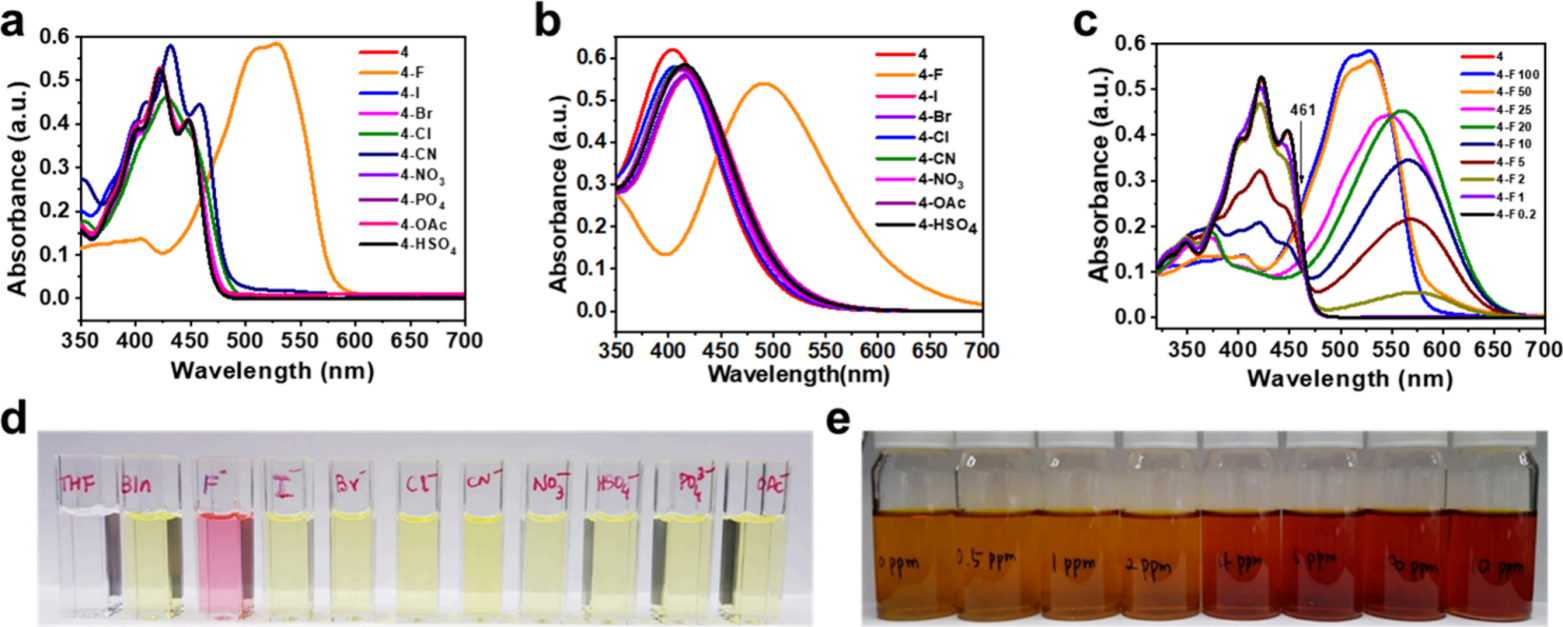
(a) Experimental and (b) theoretical UV–vis spectra tested from various anions on compound **4**. Compound **4** showed a selective absorption shift for F^–^. (c) UV–vis spectra for the limit of detection of compound **4**. Compound **4** has a 2 ppm detection limit (the numbers after F indicate the F^–^ concentration; e.g., F100 means 100 ppm of fluoride). (d) Selective visual color change of compound **4** for F^–^ (*Bln* refers to the solution of compound **4** without other anions in THF). (e) Color change of compound **4** in the aqueous solution. The trend of color change was detected with 0–10 ppm fluoride solution [the aqueous solution was made from water and DMSO at a 7:3 (v/v) ratio].

The validation of the fluoride sensing capability did encourage the evaluation of the selectivity. For that, compound **4** was tested with a variety of anions (F^–^, I^–^, Br^–^, Cl^–^, CN^–^, NO_3_^–^, PO_4_^3–^, OAc^–^, and HSO_4_^–^), and it clearly displayed a highly selective absorption behavior toward F^–^ (panels a, b, and d of Figure 3). The colorimetric change discussed above was observed only with F^–^ ions. Other anion solutions were examined without any apparent transition observed. It is important to mention that CN^–^ and OAc^–^ present the biggest challenges in F^–^ detection because they possess similar basicity and sizes and are, hence, difficult to differentiate.^18^ Strikingly, compound **4** was able to distinguish F^–^ from these competing ions.

### Limit of Detection

A critical metric that determines the efficiency of a sensor is the detection limit. With the benchmark levels of 2 and 4 ppm set by the U.S. EPA, compound **4** was evaluated against these milestones (Figure 3c). A range of fluoride concentrations from 0.2 to 100 ppm was tested to probe the detection threshold. The intensity of the absorbance showed proportional tendency with regard to the concentration of fluoride in solution. An isosbestic point was observed at 461 nm. More importantly, the recognition range was identified as 2–100 ppm because no change was observed with 1 and 0.2 ppm. In short, the limit of detection for compound **4** was evaluated as 2 ppm. In addition, the detection in aqueous media was also recorded (Figure 3e). The fluoride concentration range for this experiment was 0–10 ppm, and color change also appeared to be noticeable to the naked eye. As depicted in the figure, the limit of detection was determined to be 4 ppm.

### Theoretical Study

**Figure 4:**
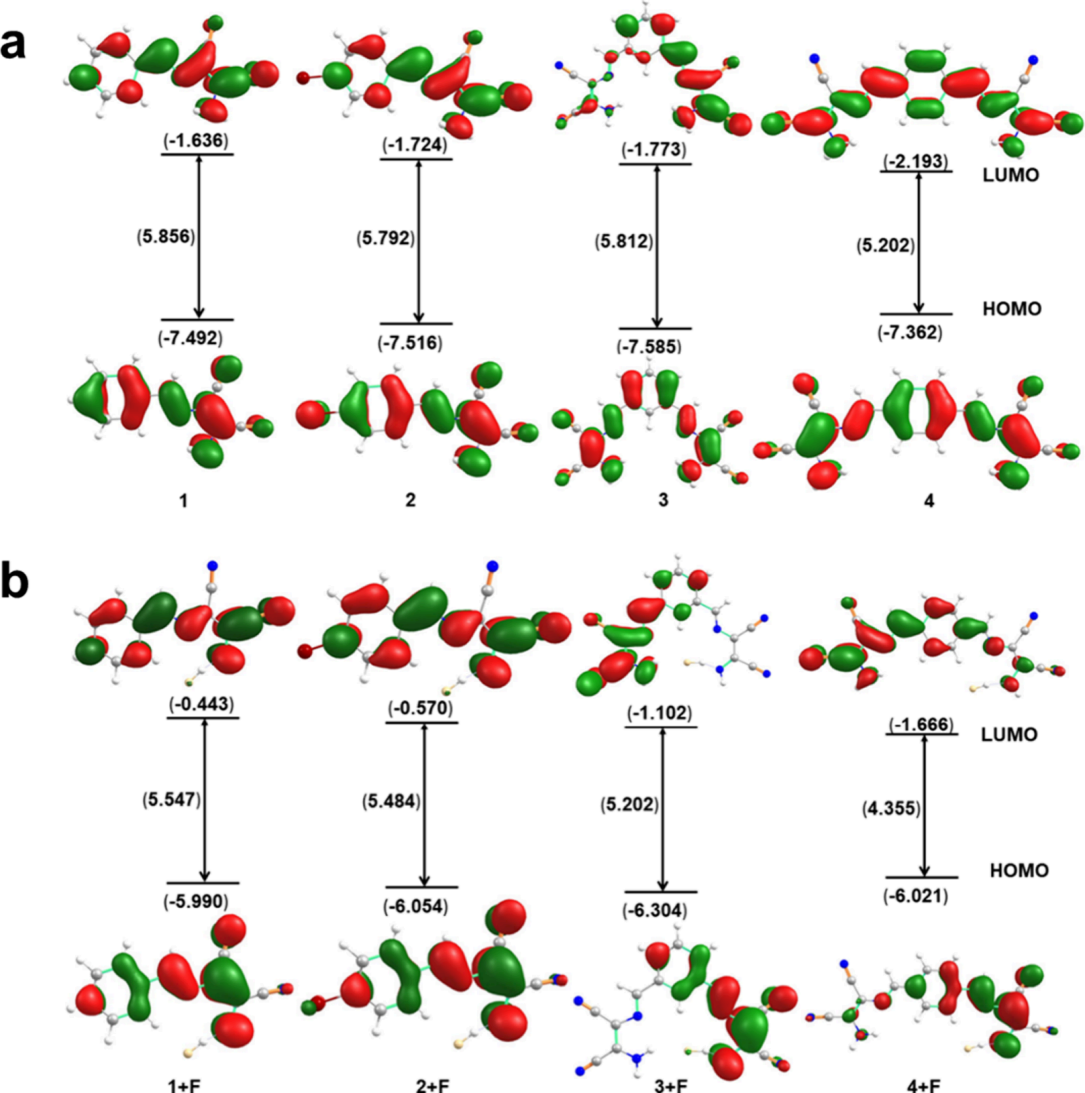
Frontier molecular orbital analysis of (a) compounds **1**, **2**, **3**, and **4** and (b) compounds **1** + F, **2** + F, **3** + F, and **4** + F calculated at the CAM-B3LYP-D3/6-311++G(d,p)-CPCM level (solvent = THF).

The frontier molecular orbital analysis showed that the band gap could be altered with the substitution of the proton by bromine (compounds **1** and **2**) as a result of the inductive effect (Figure 4a). However, this band gap distortion was more prominent in the case of compounds **3** and **4**, where the position of the π conjugation was changed from *ortho* to *para*. The observed difference in the band gap for compounds **1** and **2** was determined to be 0.064 eV, while for compounds **3** and **4**, it was 0.610 eV, and this greater change emphasized the significance of the resonance effect. The highest occupied molecular orbital (HOMO)–lowest unoccupied molecular orbital (LUMO) and band gap energies of compounds **1** + F, **2** + F, **3** + F, and **4** + F were also calculated to seek correlation with the observed experimental colorimetric conversion. As expected, the greatest HOMO–LUMO gap narrowing was computed to be that of the transition from compound **4** to compound **4** + F, with a value of 0.847 eV (Figure 4b). This indicated an extensively stabilizing interaction between fluoride and compound **4** that resulted in the augmentation of the π electron delocalization. Such an interaction should go beyond a simple hydrogen-bonding event because hydrogen bonding would not bring about a dramatic electronic shift. The moderate basicity of fluoride could presumably allow it to exert proton transfer with the hydrogen atoms on the free amino groups, enabling heightened conjugation (Figure S10 of the Supporting Information).

**Figure 5:**
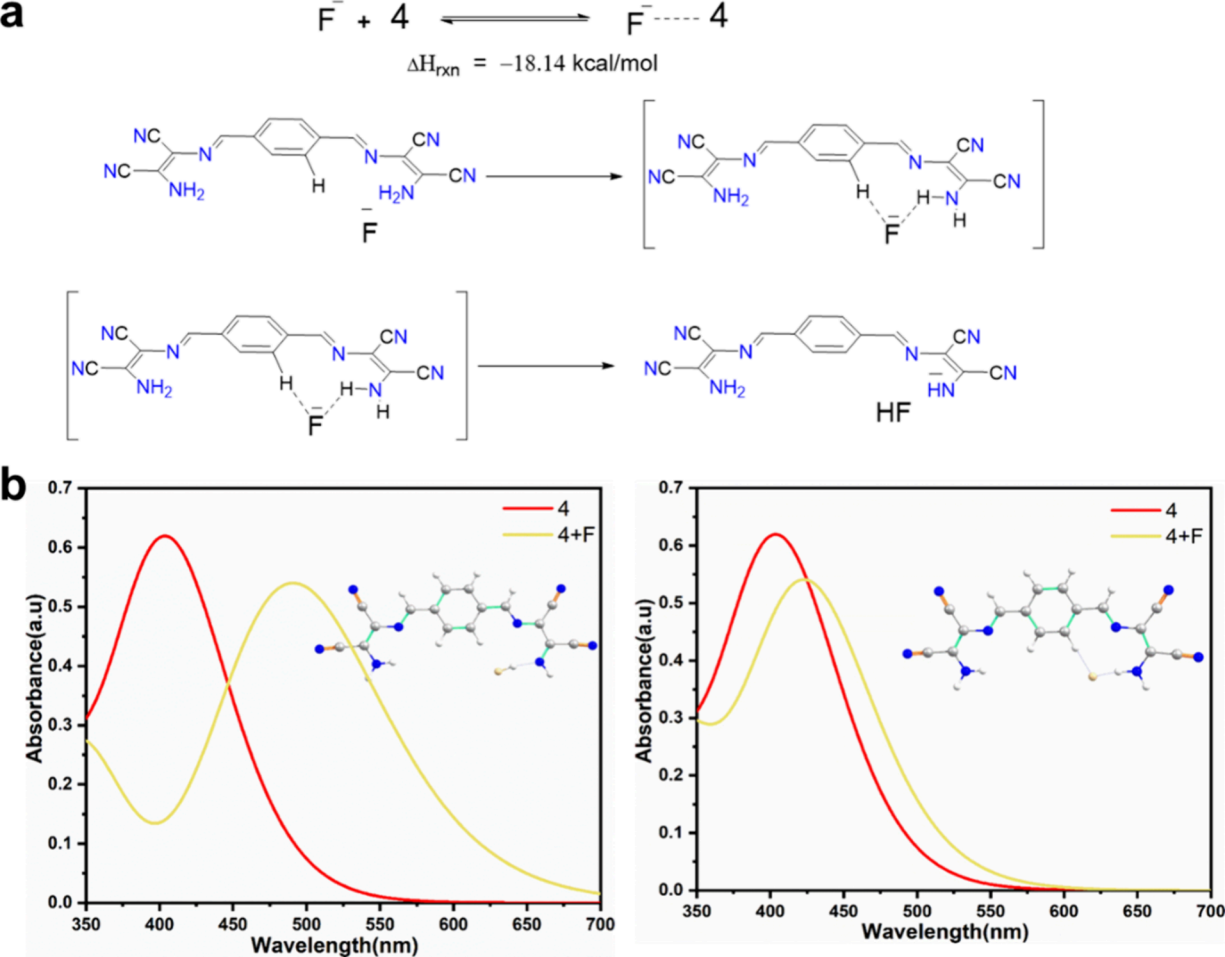
(a) Enthalpy change for the deprotonation of compound **4** by F^–^ calculated at the B3LYP/6-311+G(d,p)-CPCM level (solvent = THF). (b) UV–vis spectra were calculated at the CAM-B3LYP/6-311G++(d,p)-CPCM level (solvent = THF).

The deprotonation scenario was theoretically examined in which fluoride was initially attracted by the force of hydrogen bonds with the protons on the sensor molecule and subsequently underwent deprotonation (Figure 5a). The overall heat of reaction was calculated to be −18.14 kcal/mol, indicating an exothermic process. Furthermore, the simulated UV–vis spectrum of the deprotonation complex (left panel of Figure 5b) showed a greater resemblance to the experimental spectrum than that of the mere hydrogen-bonding complex (right panel of Figure 5b).

**Figure 6:**
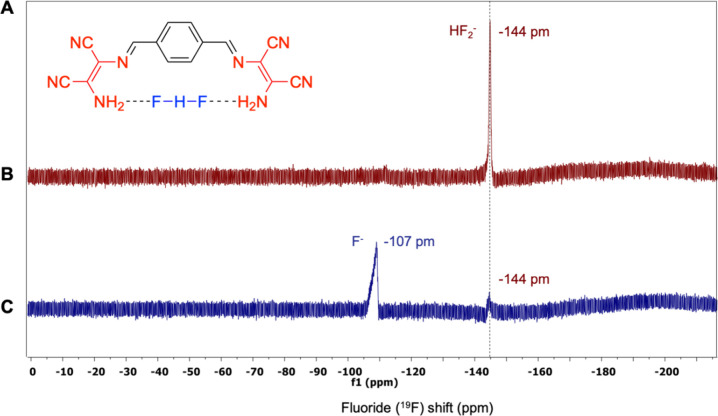
(a) Plausible compound **4** + F interaction. (b) HF_2_^–^ peak in ^19^F NMR at −147 ppm. (c) F^–^ peak from TBAF in ^19^F NMR at −107 ppm.

To gain more insight into the interaction between fluoride and compound **4**, a ^19^F NMR spectra study was carried out (Figure 6). It was observed that the spectrum of the TBAF solution showed a signal for free F^–^ at −107 ppm. However, upon addition of compound **4**, a completely distinct peak arose at −147 ppm, replacing the free F^–^ peak. This signal is characteristic of the HF_2_^–^ species. In fact, it also appeared in the TBAF spectrum as a minor peak as a result of the presence of water (Figure 6c). Nonetheless, the intensity of the signal was much more pronounced in the ^19^F NMR spectrum of [**4** + F^**–**^]. Considering that the solvent of the whole system was still THF, this result indicated that HF_2_^–^ species existed in a higher concentration and more stabilized form, enabled by the complexation with compound **4**. The plausible structure was proposed in Figure 6a, in which the fluoride atoms were symmetrically located in a hydrogen-bonding immobilization mode with the amino group protons.

## Conclusion

We have developed a cheap, effective, and highly selective fluoride ion sensor, constructed from two commercially available reagents, terephthalaldehyde and DAMN. The synthetic preparation was easily scaled up to 30 g in a rapid reaction and facile purification via methanol washing. This made the compound among the most accessible molecular fluoride sensors by far. Systematic structural investigation uncovered the role of electron-withdrawing groups in tuning the visible light absorption of the sensor toward the visual range. In addition, the *para* substitution pattern of the DAMN units facilitated the elongation of π electron conjugation that would be emboldened upon the deprotonating interaction with fluoride. ^19^F NMR spectra revealed the presence of stabilized HF_2_^–^ in the complex with the sensor molecule through hydrogen bonding. Such assembly revealed a red color that was in stark contrast with the yellow sensor, allowing for a clear recognition by the naked eye. Detection limits were determined as 2 and 4 ppm in THF and water, respectively. These thresholds closely matched the levels recommended by the U.S. EPA for fluoride contents in drinking water. Incorporation of the developed sensor into a field application is underway.

## Supporting Information Available

Theoretical methodology, ^1^H and ^19^F NMR spectra, FTIR spectra, simulated isosurfaces, table of reported sensors, and Cartesian coordinates of the optimized geometries (PDF)
