# Supplementary material for: Highly Selective and Scalable Molecular Fluoride Sensor for Naked-Eye Detection
Source: ACS Appl Mater Interfaces. 2024 Mar 30;17(12):17767–74. doi: 10.1021/acsami.4c01187 (PMC11955946; doi:10.1021/acsami.4c01187)
Supplement: Supplementary file 1 — am4c01187_si_001.pdf [file am4c01187_si_001.pdf]

Supporting Information (SI) for

Highly selective and scalable molecular fluoride sensors for naked-eye detection

Zakir Ullah[1,‡], Saravanan Subramanian,[2,‡] Haeseong Lim,[3] Nesibe A. Dogan,[4] Joo Sung Lee,[4] Thien S. Nguyen*[5], Cafer T. Yavuz*[5]

[1] Institut de Ciència de Materials de Barcelona (ICMAB-CSIC), Consejo Superior de Investigaciones Científicas, Campus Universitari de Bellaterra, Cerdanyola del Vallès 08193, Spain.
[2] Inorganic Materials and Catalysis Division, CSIR-Central Salt and Marine Chemicals Research Institute, Bhavnagar, Gujarat, India
[3] Department of Materials Science and Engineering, KAIST, 291 Daehak-ro, Yuseong-gu, Daejeon, 34141, Republic of Korea
[4] Department of Chemical and Biomolecular Engineering, KAIST, Daejeon, 34141 Korea
[5] Oxide and Organic Nanomaterials for Energy and Environment (ONE) Lab, Chemistry Program, Advanced Membranes & Porous Materials Center, KAUST Catalysis Center, Physical Science & Engineering (PSE), King Abdullah University of Science and Technology (KAUST), Thuwal 23955, Saudi Arabia
‡ These authors contributed equally.
* Correspondence: sithien.nguyen@kaust.edu.sa (T.S.N.) cafer.yavuz@kaust.edu.sa (C.T.Y.)

TABLE OF CONTENTS

A. Supporting Methods S2

B. Supporting Figures S3

C. Supporting Tables S13

A. Supporting Methods

Theoretical Methodology

All electronic structure calculations were carried out using density functional theory (DFT) with a hybrid functional B3LYP together with DFT-D3 method by Grimme for considering dispersion interactions[1], and well-accepted basis sets: 6-311+G** and 6-31++G** as implemented in the Gaussian 16[2]. All the calculations were performed at the default temperature and pressure (298.15 K and 1.00 atm). Conductor-like polarizable continuum model (CPCM) method were used to apply the solvent effects in the calculations. Furthermore, the frequency calculations have been carried out to confirm the nature of obtained minima at the same level of theory as the geometry optimization. The Uv-vis spectra simulations were performed at the time dependent (TD), TD-CAM-B3LYP/6-311++G** level of theory[3]. To analyze the real space functions and characterize the type of the different interaction we conduct the quantum theory of atoms in molecules (AIM)[4] using the Multiwfn software with the wave functions generated from the Gaussian 16[5]. The topology of FSen-4-F, FSen-4-CN, FSen-4-OAc interactions were analyzed according to the Bader's AIM theory, using the Multiwfn code[5]. The nature and strength of the interactions were displayed in the isosurface.

B. Supporting Figures

1. ^{1}H NMR

1.1. Structure **1**

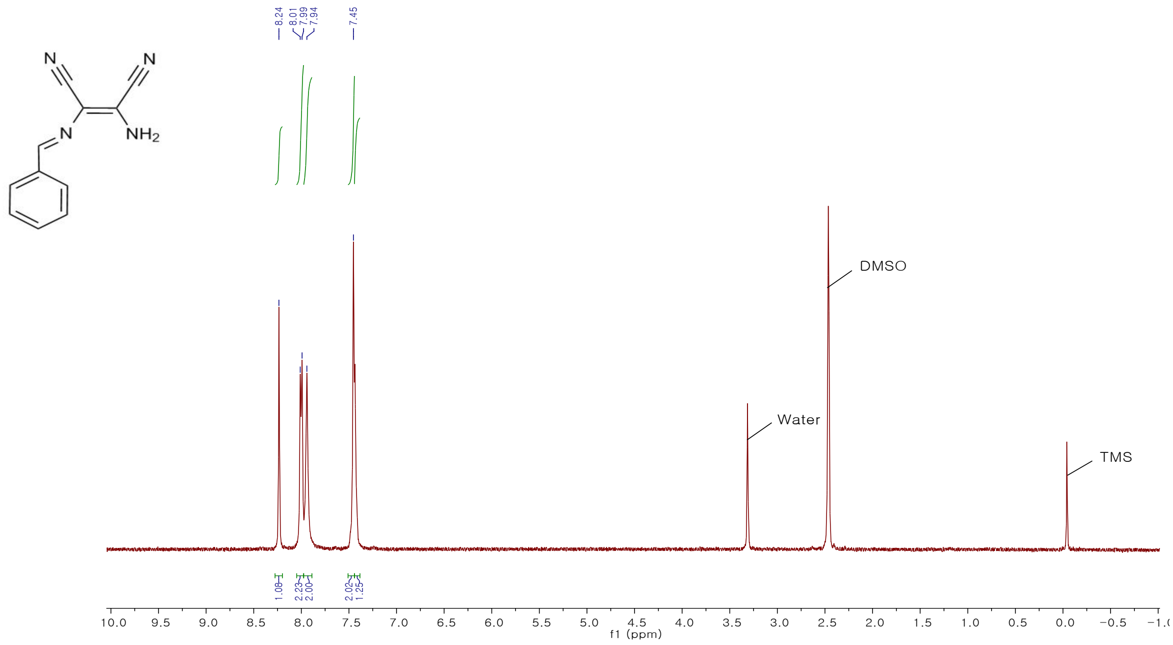

Figure S1. ^{1}H NMR spectrum of **1**.

1.2. Structure **2**

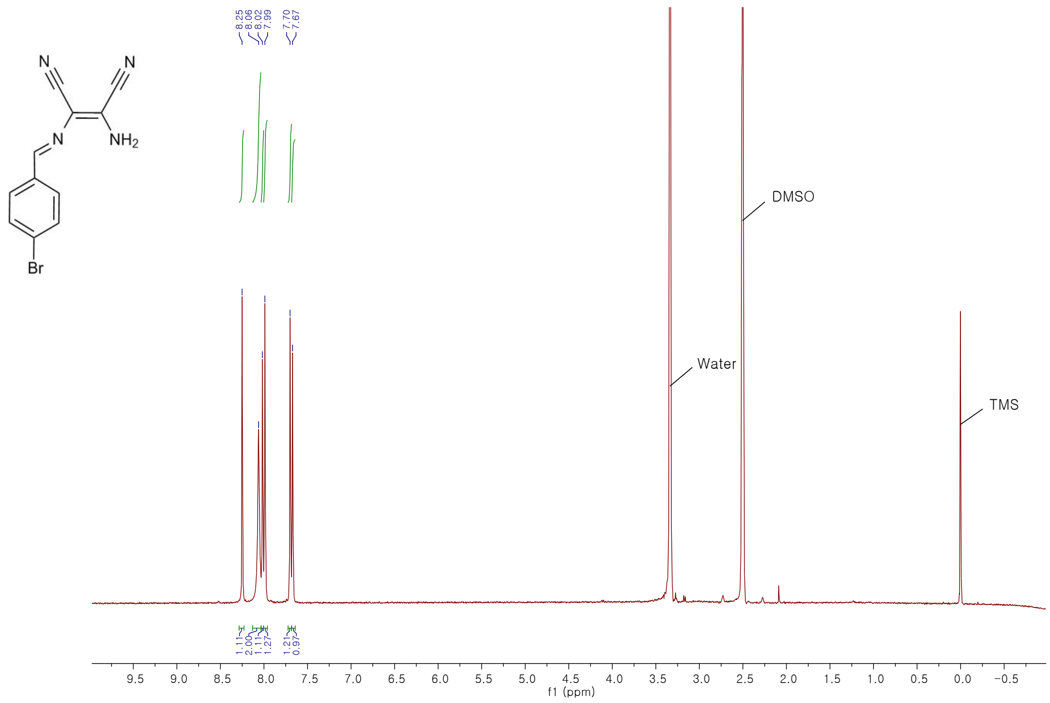

Figure S2. ^{1}H NMR spectrum of **2**.

1.3. Structure **3**

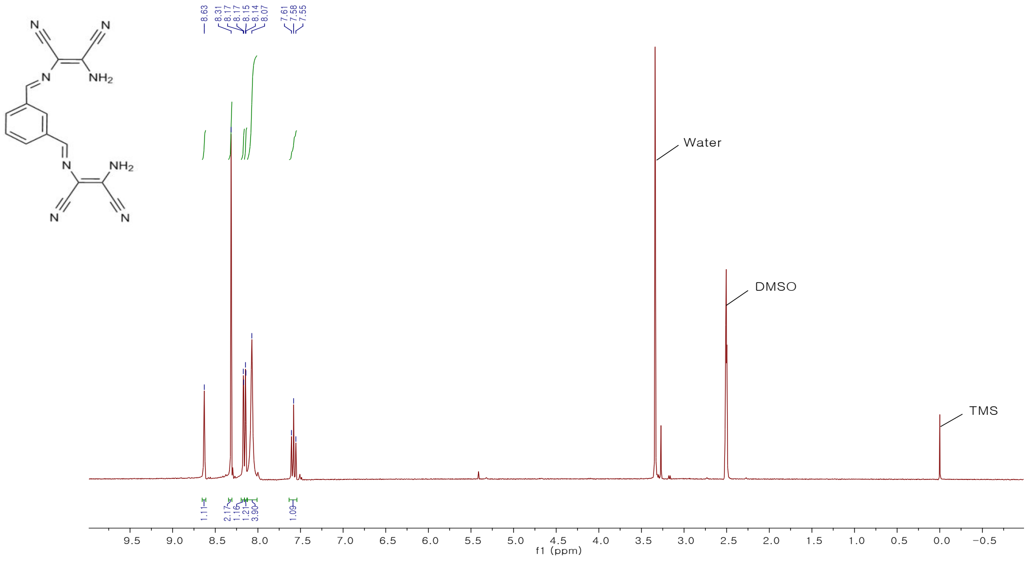

Figure S3. ^{1}H NMR spectrum of **3**.

1.4. Structure **4**

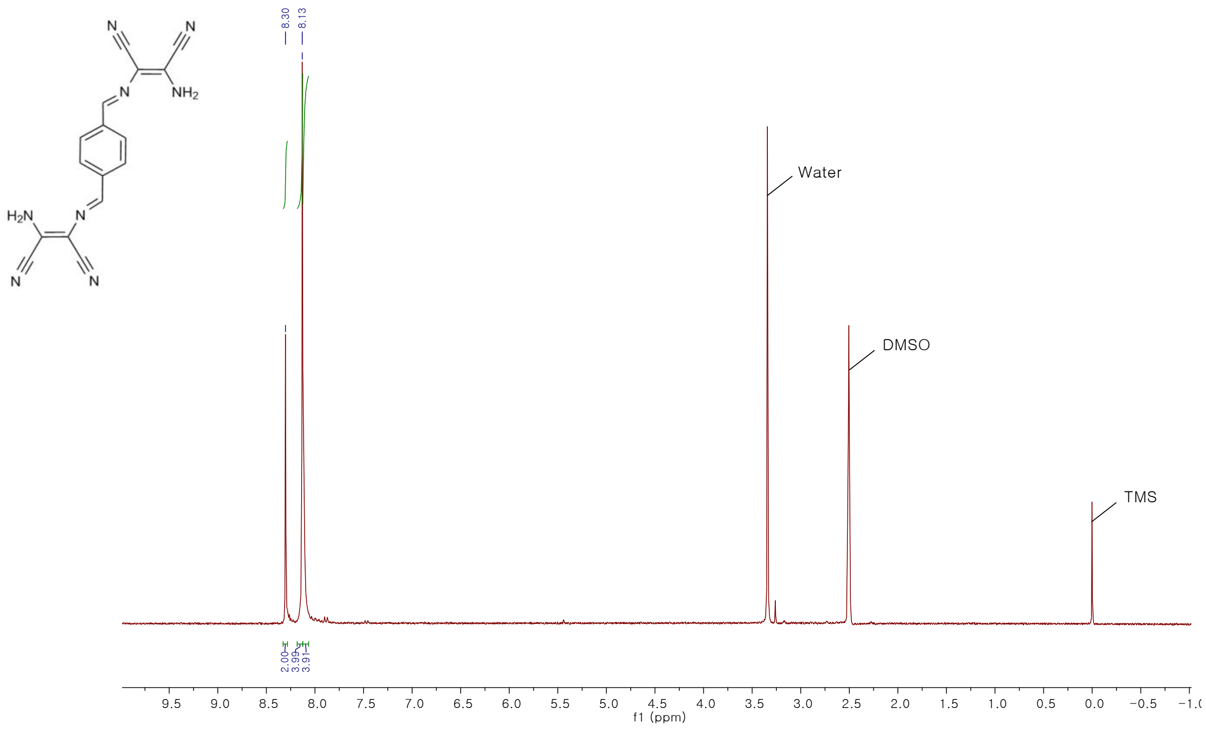

Figure S4. ^{1}H NMR spectrum of **4**.

2. ^{19}F NMR

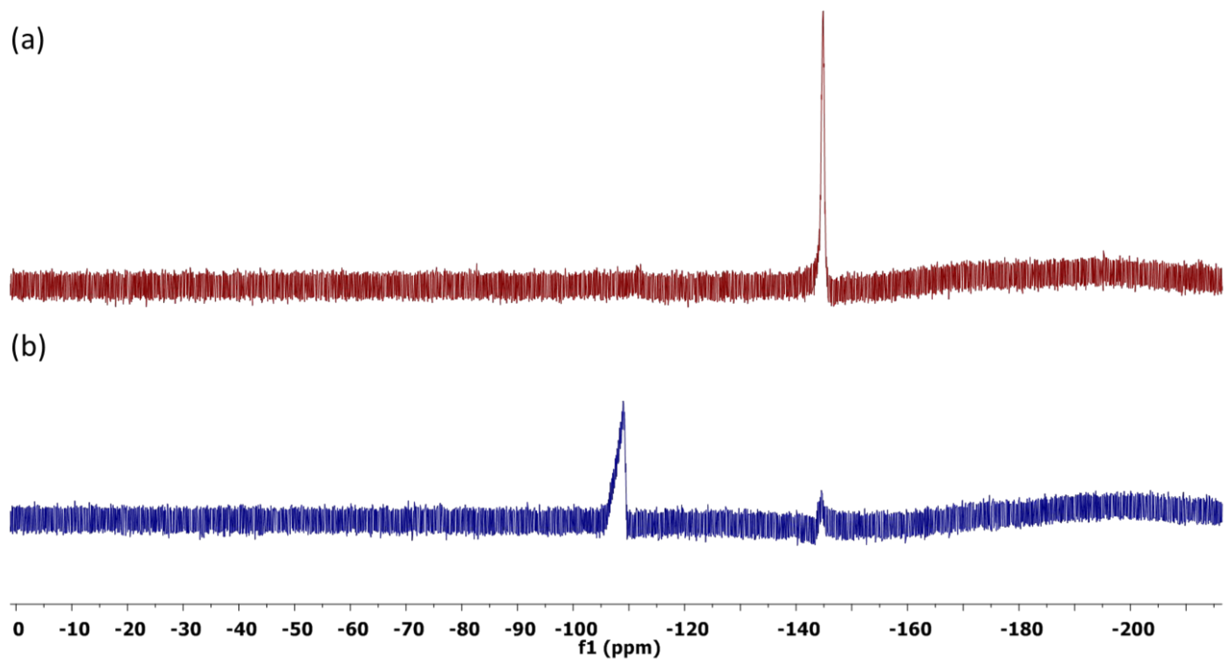

Figure S5. ^{19}F NMR Spectra of **4** and **[4+F] complex**. (a) HF^{2-} peak in 19F NMR at -147 ppm. (b) F^{-} peak from TBAF in ^{19}F NMR at -107 ppm.

3. FT-IR

3.1. Structure **1**

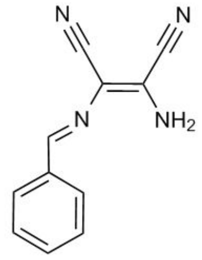

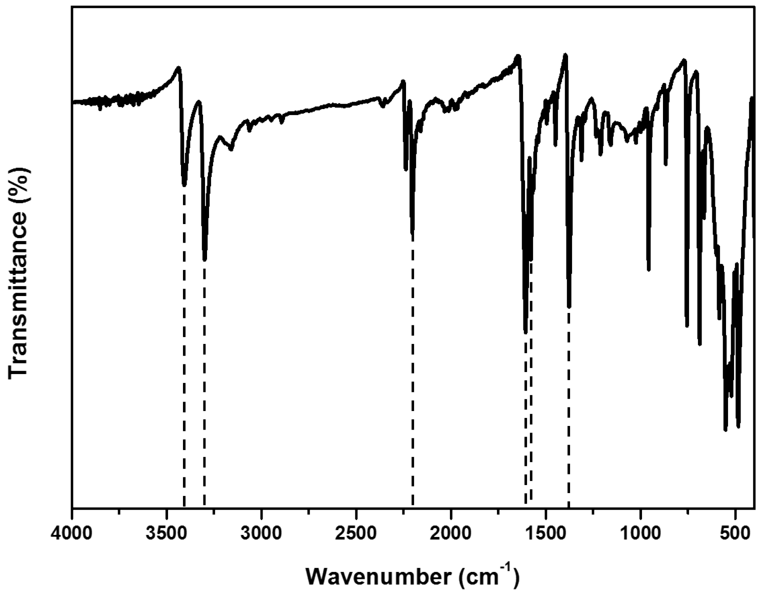

Figure S6. FT-IR spectrum of **1**.

3.2. Structure **2**

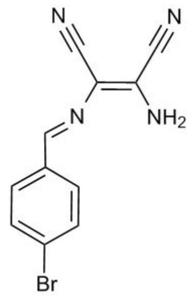

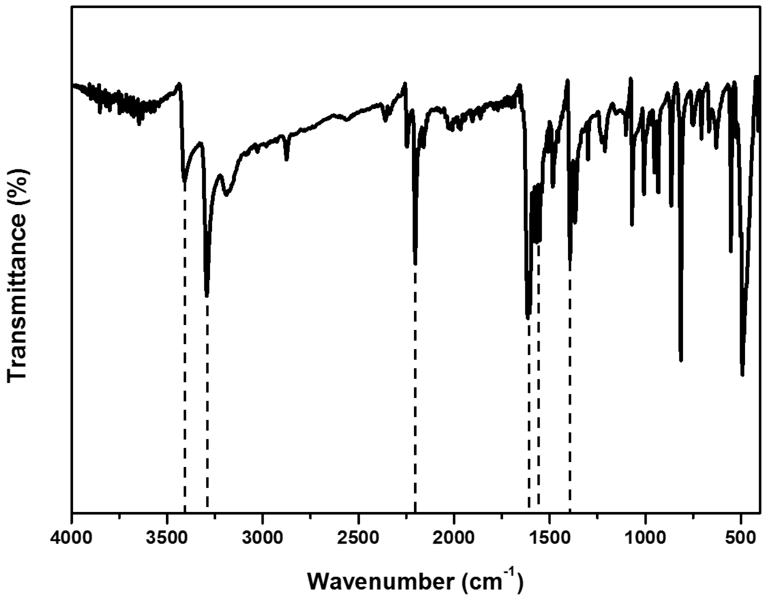

Figure S7. FT-IR spectrum of **2**.

3.3. Structure **3**

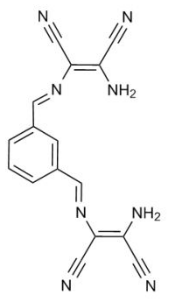

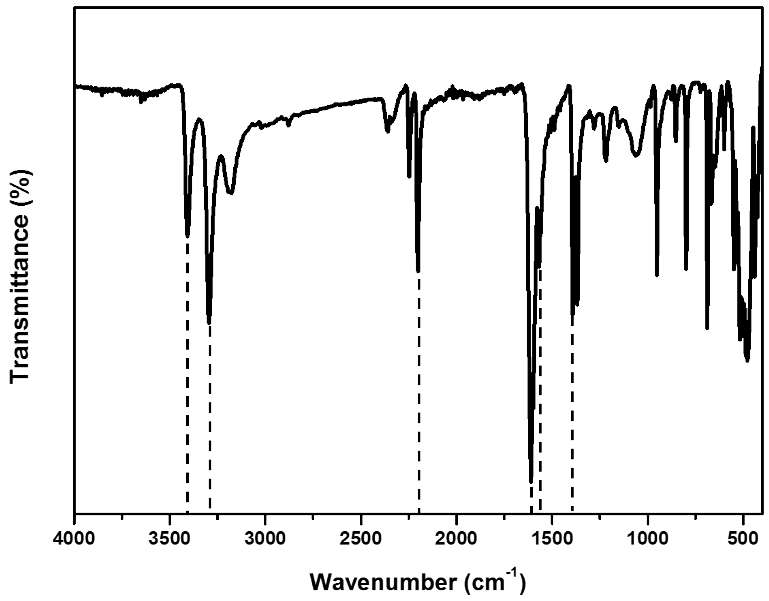

Figure S8. FT-IR spectrum of **3**.

3.4. Structure **4**

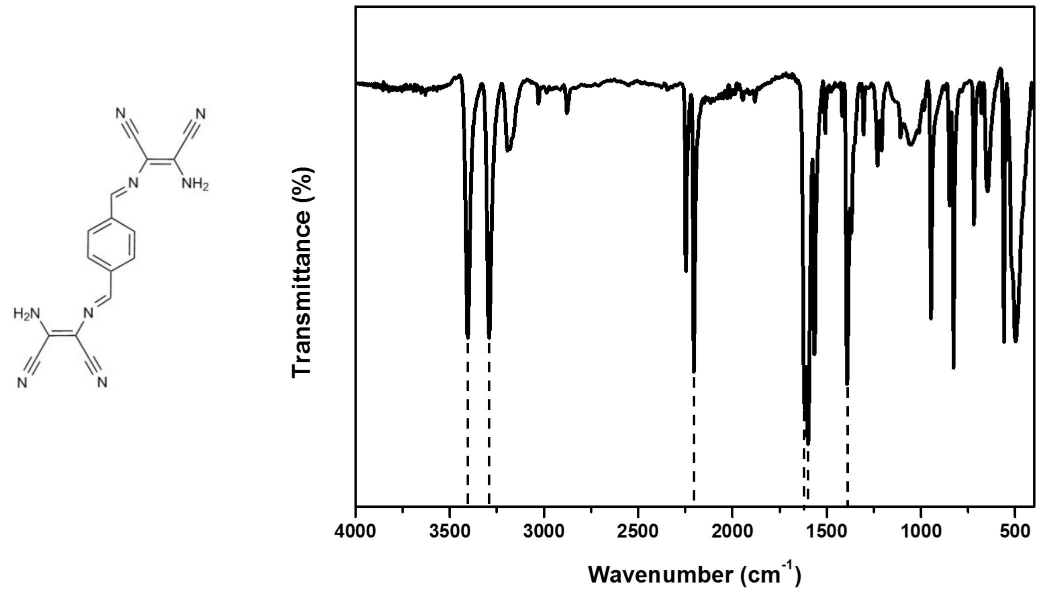

Figure S9. FT-IR spectrum of **4**.

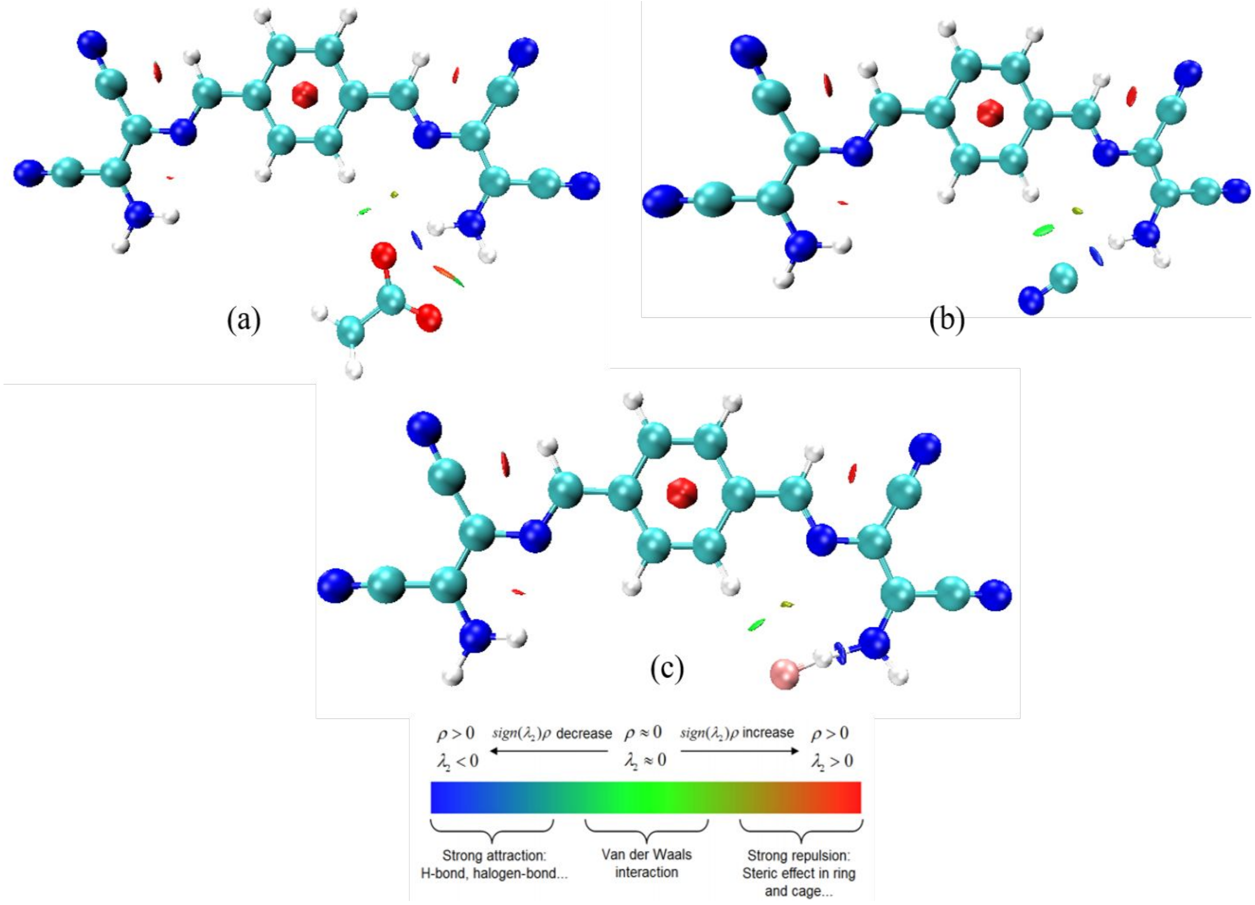

Figure S10. Simulated isosurfaces for the a) **4**+OAc, b) **4**+CN, and c) **4**+F.

Figure S10 display the type of interactions of the **4** and with the corresponding anions OAc^{-}, CN^{-}, and F^{-} respectively. Both the nitrile (CN) and acetate (OAc) shows strong H-bonding with the **4**, however the fluoride anion shows the deprotonation mechanism and that resulting HF form has strong H-bonding with the **4**.

C. Supporting Tables

Table S1. Previously reported study about anion detections.

Reference	Naked eye detection	Recyclability	Cost-$	Surfactant	CN Selection	F-Concentration	Solvent	HF2-	Detection limit	Materials
[6] Jeyanthi-SpecChimA-2015-indolediamino	yes	no	>50	no	yes	0.5 equiv. from TBAF in DMSO	DMSO	no	0.07ppm	2,3-bis((E)-(1H-indole-3-yl)methyleneamino)maleonitrile 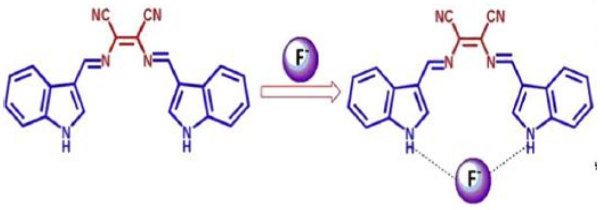
[7] Hinterholzinger-SciRep-2013-mofinwater	no	No	>100	No	No/not tried	No naked eye detection	water	Did F nmr	0.015 ppm	a porous crystalline framework serves as a host for a fluorescent molecule. 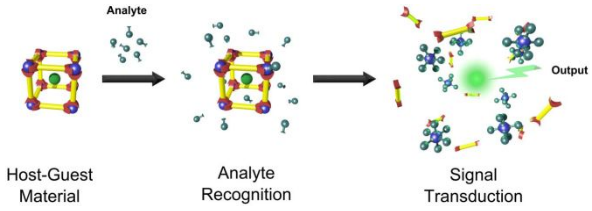
[8] Chavali-AnalCheRes-2015-potable water	no	No	>30	no	No/not tried	NaF in water/	water	no	0.2ppm	7-O-tertbutyldiphenylsilyl-4-methylcoumarin (TBDPSC) that releases fluorescent molecules 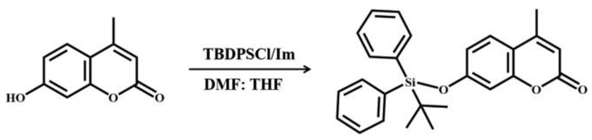

[9]Boxi-DaltonTrans-2015-CdSZnSnanoparticles	[10]Ashokkumar-Angew-2013-Test	[11]Sarkar-Dalton-2016-anthraimidazoledione	Reference	[12]Miyaji-Angew-2001-anionsens
no	yes	Yes with Cu+2	Naked eye detection	yes
No	no	no	Recyclability	No
>100	>50	>50	Cost-$	>100
No	9mM MES buffer	no	Surfactant	no
No/not tried	No/not tried	Only in the presence of Cu+2	CN selection	No/not tried
30ppm of salts for selectivity	Addition of NaF salt (0–0.8 ppm) in	6 equiv of anions	F-concentration	F- in water solution 1 ppm
water	50% aqueous solution	acetonitrile and	Solvent	Dissolve in Acetonitrile-paper-dip in water
no	no	yes	Hf2-	no
0.1 ppm	0.01ppm	0.04 ppm	Detection limit	1ppm
Ag-CdS/Ag-ZnS core/shell Nanoparticle- L-cysteine capped 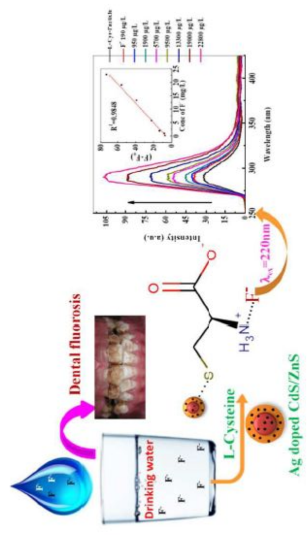	BODIPY–amidothiourea dyes 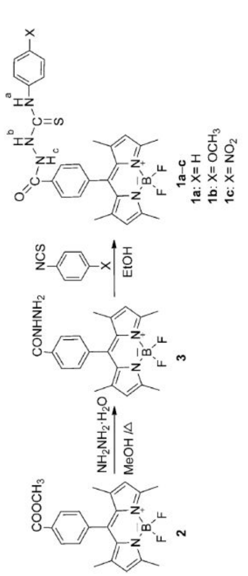	four anthraimidazoledione compounds 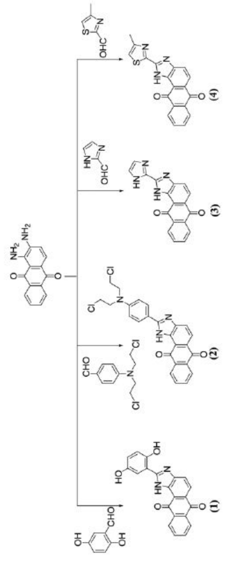	Materials	Ru-bipy fluorophore (bipy = 2,2-bipyridine) and a 2,4-dinitrophenylhydrazone 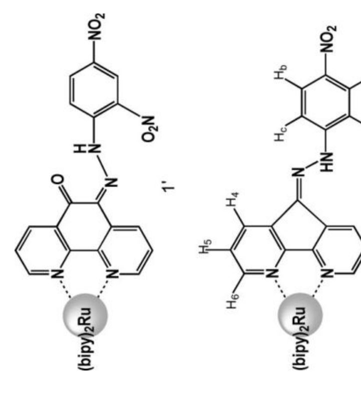 1' 1 chromophore

[13]Zhang-2018-CheSci-azabora	[14]Psent-Lopez-Alled-ChComm-2017-Azulene	[15]Oshchepkov-JOChem-2018-macrocycle	[16]Lin-CheComm-2006-inwaterdetection	[17]Kim-Gabbai-JACS-2009-water4ppm
No	Yes dilute	No	yes	no
no	no	no	no	no
>100	>100	>50	>200	>100
no	water/CTAB/TBAS/Az-1-Bpin	pH buffer	No	no
Yes	No/not tried	No/not tried	No/not tried	No/not tried
receptor concentration of 1*10-5	3.4 mg/L	50 equiv of anions in 10 mM MES buffer, pH	F- in water solution 10 ppm	10 mM pyridine buffer, pH 4.9)
THF	Water/surfactant	Aqueous buffer solution	Dissolve in Acetonitrile-paper-dip in water	buffer pH 4.9, water
no	no	no	no	no
Not mentioned	3.4 ppm	Not mentioned	10ppm	1.9 ppm
polymerizable 9,10-azaboraphenanthrene (BNP)-containing monomers 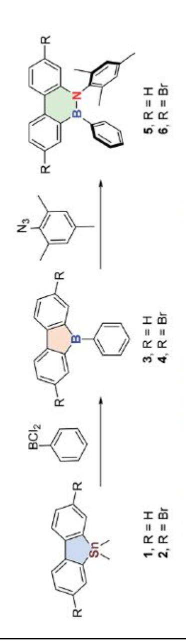	Azulene–boronate esters 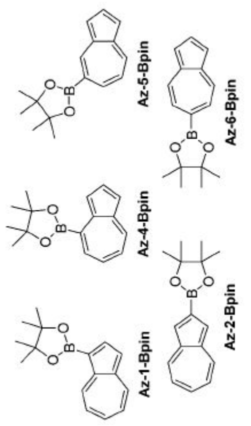	Hybrid Macrocycles 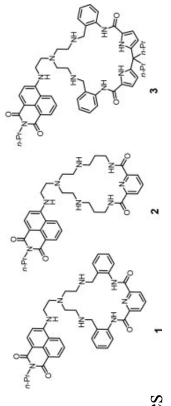	Ru-bipy based quinonehydrazone 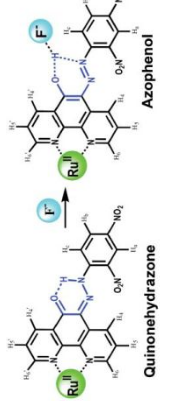	phosphonium boranes 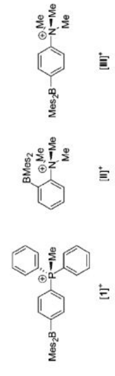

reference	[18]Kaloo-Analyst-2013-hf2diamino	[19]Cho- ORGANIC LETTERS-2005-Visible	[20]Bartocci-DyesPigm-2017-diamino	[21]ang-JPorPhtalo-2010-porphyrin
Naked eye detection	yes	yes	yes	optical change almost never occured
Cyclability	No	no	no	no
Cost-$	>20	>50	>100	>100
Surfactant	No	no	no	no
CN selection	Not highly selective	No/not tried	No/not tried	No/not tried
F-concentration	All Tba salts	TBAF (3 x 10^{-3} M) in DMSO	TBAF (5 x 10^{-5} M) in chloroform	TBAF (3.5 x 10^{-3} M) in chloroform
Solvent	THF, DMSO	DMSO	Chloroform	Chloroform
Hf2-	yes	no	no	no
Detection limit	Not mentioned	Not mentioned	6.5ppm	Not mentioned
Material	2-Amino-((E)-(4-cyanobenzalidine)amino)maleonitrile 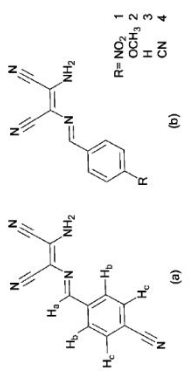	A nitro group and an azo unit into an aromatic moiety 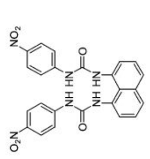	uranyl-N,N0-bis(3,5-ditertbutyl-2-hydroxybenzylidene)-1,2-dicyano-1,2-ethenediamine 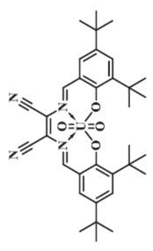	tetrapyrazinoporphyrazine 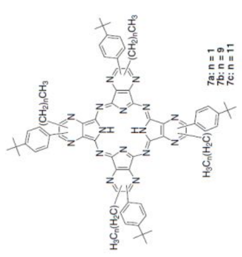

[22]Jo-NJChe-2015-diamino	[23]Khanmohammadi-JMolSens-2017-diamino	reference	[24]Uahengo-Jfluor-2017-DMSO	[25]Kim-OrgLett-2008-chromogenic	26Sokkalingam-JorgChe-2011-Highly
yes	yes	Naked eye detection	yes	yes	yes
yes	no	Cyclability	no	no	no
>50	>50	Cost-$	>50	>50	>100
2.997 mL of DMSO/ [illegible]	no	Surfactant	no	no	no
yes	no	CN selection	Not mentioned	Not mentioned	yes
600 equiv. from	5 equiv. from TBAF	F-concentration	30 equiv.	2 equiv. from TBAF	1 equiv. of TBAF
DMSO	CH_3CN	Solvent	DMSO	CH_3CN/H_2O (50:50, v/v)	CH_3CN/H_2O
no	no	Hf2-	no	no	no
219ppm	10.4ppm	Detection limit	Not mentioned	Not mentioned	50 nM
Synthesis by 2,3-diaminomaleonitrile and julolidine moiety 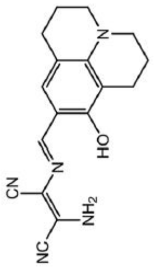	diaminomaleonitrile-based azo dyes 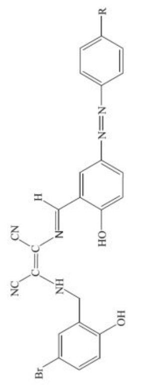	Material	2,2′-dinaphtholazobenzene molecular framework 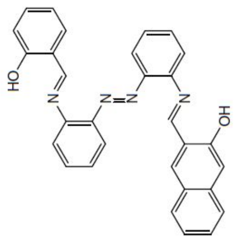	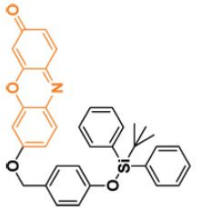	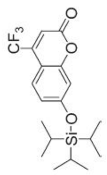

27Hu-Angew-2010-A Rapid	Yes	yes	>100	CTAB	no	1 equiv. of NaF	THF:H_2O(with CTAB)	no	100 ppb	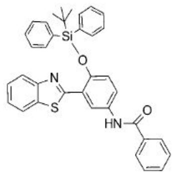

References

1. (a) Lee, C.; Yang, W.; Parr, R. G., Development of the Colle-Salvetti correlation-energy formula into a functional of the electron density. *Phys. Rev. B* **1988,** *37* (2), 785; (b) Becke, A. D., Density-functional exchange-energy approximation with correct asymptotic behavior. *Phys. Rev. A* **1988,** *38* (6), 3098.
2. (a) Frisch, M.; Trucks, G.; Schlegel, H.; Scuseria, G.; Robb, M.; Cheeseman, J.; Scalmani, G.; Barone, V.; Petersson, G.; Nakatsuji, H., Gaussian 16. *Revision A* **2016,** *3*; (b) Frisch, M.; Trucks, G.; Schlegel, H.; Scuseria, G.; Robb, M.; Cheeseman, J.; Scalmani, G.; Barone, V.; Petersson, G.; Nakatsuji, H., Gaussian 16, Revision A. 03, Gaussian. *Inc., Wallingford CT* **2016**.
3. Bernini, C.; Zani, L.; Calamante, M.; Reginato, G.; Mordini, A.; Taddei, M.; Basosi, R.; Sinicropi, A., Excited state geometries and vertical emission energies of solvated dyes for DSSC: a PCM/TD-DFT benchmark study. *J. Chem. Theory Comput.* **2014,** *10* (9), 3925-3933.
4. Bader, R. F., Atoms in molecules. *Acc. Chem. Res.* **1985,** *18* (1), 9-15.
5. Lu, T.; Chen, F., Multiwfn: a multifunctional wavefunction analyzer. *J. Comput. Chem.* **2012,** *33* (5), 580-592.
6. Jeyanthi, D.; Iniya, M.; Krishnaveni, K.; Chellappa, D. Novel indole based dual responsive "turn-on" chemosensor for fluoride ion detection. *Spectrochim. Acta A* **2015**, *136*, 1269-74.
7. Hinterholzinger, F. M.; Ruhle, B.; Wuttke, S.; Karaghiosoff, K.; Bein, T. Highly sensitive and selective fluoride detection in water through fluorophore release from a metal-organic framework. *Sci. Rep.* **2013**, *3*, 2562.
8. Chavali, R.; Gunda, N. S. K.; Naicker, S.; Mitra, S. K. Rapid detection of fluoride in potable water using a novel fluorogenic compound 7-O-tert-butyldiphenylsilyl-4-methylcoumarin. *Anal. Chem. Res.* **2015**, *6*, 26-31.
9. Boxi, S. S.; Paria, S. Fluorometric selective detection of fluoride ions in aqueous media using Ag doped CdS/ZnS core/shell nanoparticles. *Dalton Trans.* **2016**, *45* (2), 811-9.
10. Ashokkumar, P.; Weisshoff, H.; Kraus, W.; Rurack, K. Test-strip-based fluorometric detection of fluoride in aqueous media with a BODIPY-linked hydrogen-bonding receptor. *Angew. Chem., Int. Ed.* **2014**, *53* (8), 2225-9.

11. Sarkar, A.; Bhattacharyya, S.; Mukherjee, A. Colorimetric detection of fluoride ions by anthraimidazoledione based sensors in the presence of Cu(ii) ions. *Dalton Trans.* **2016,** *45* (3), 1166-75.
12. Miyaji, H.; Sessler, J . L. Off-the-Shelf Colorimetric Anion Sensors. *Angew. Chem., Int. Ed.* **2001**, *40* (1), 154-157.
13. Zhang, W.; Li, G.; Xu, L.; Zhuo, Y.; Wan, W.; Yan, N.; He, G. 9,10-Azaboraphenanthrene-containing small molecules and conjugated polymers: synthesis and their application in chemodosimeters for the ratiometric detection of fluoride ions. *Chem. Sci.* **2018**, *9* (19), 4444-4450.
14. Lopez-Alled, C. M.; Sanchez-Fernandez, A.; Edler, K. J.; Sedgwick, A. C.; Bull, S. D.; McMullin, C. L.; Kociok-Kohn, G.; James, T. D.; Wenk, J.; Lewis, S. E. Azulene-boronate esters: colorimetric indicators for fluoride in drinking water. *Chem. Commun.* **2017**, *53* (93), 12580-12583.
15. Oshchepkov, A. S.; Shumilova, T. A.; Namashivaya, S. R.; Fedorova, O. A.; Dorovatovskii, P. V.; Khrustalev, V. N.; Kataev, E. A. Hybrid Macrocycles for Selective Binding and Sensing of Fluoride in Aqueous Solution. *J. Org. Chem.* **2018**, *83* (4), 2145-2153.
16. Lin, Z. H.; Ou, S. J.; Duan, C. Y.; Zhang, B. G.; Bai, Z. P. Naked-eye detection of fluoride ion in water: a remarkably selective easy-to-prepare test paper. *Chem. Commun.* **2006**, (6), 624-6.
17. Gabbaı, Y. K. a. F. P. Kim-Gabbai. Cationic Boranes for the Complexation of Fluoride Inons in Water below the 4 ppm Maximum Contaminant Level. *J. Am. Chem. Soc.* **2009**, *131* (9), 3363–3369.
18. Kaloo, M. A.; Sankar, J. Exclusive fluoride ion recognition and fluorescence "turn-on" response with a label-free DMN Schiff base. *Analyst.* **2013**, *138* (17), 4760-3.
19. Cho, E. J; Ryu, B. J.; Lee, Y. J.; Nam, K. C. Visible Colorimetric Fluoride Ion Sensors. *Org. Lett.* **2005**, *7* (13), 2607-2609.
20. Bartocci, S.; Sabaté, F.; Bosque, R.; Keymeulen, F.; Bartik, K.; Rodríguez, L.; Dalla Cort, A. Colorimetric and fluorescence “turn-on” recognition of fluoride by a maleonitrile-based uranyl salen-complex. *Dyes Pigm.* **2016**, *135*, 94-101.
21. Jang, C. K.; Kim, S. H.; Jaung, J.-Y. Synthesis and optical properties of tetrapyrazinoporphyrazines containing asymmetrical alkyl chains and t-butylphenyl groups. *J. Porphyr. Phthalocyanines.* **2010,** *14* (06), 531-539.
22. Jo, T. G.; Na, Y. J.; Lee, J. J.; Lee, M. M.; Lee, S. Y.; Kim, C., A diaminomaleonitrile based selective colorimetric chemosensor for copper(ii) and fluoride ions. *New J. Chem.* **2015**, *39* (4), 2580-2587.
23 Khanmohammadi, H.; Arab, V.; Rezaeian, K.; Talei, G. R.; Pass, M.; Shabani, N. Diaminomaleonitrile-based azo receptors: Synthesis, DFT studies and their antibacterial activities. *J. Mol. Struct.* **2017**, *1129*, 169-178.
24. Uahengo, V.; Zhang, Y.; Xiong, B.; Zhao, P.; Cai, P.; Rhyman, L.; Ramasami, P.; Hu, K.; Cheng, G. A Fluoro-Chromogenic Sensor Based on Organic Molecular Framework for Cu(2+) and F(-) in Aqueous Soluble DMSO. *J. Fluoresc.* **2017**, *27* (1), 191-197.

25. Sokkalingam, P.; Lee, C. H. Highly sensitive fluorescence "turn-on" indicator for fluoride anion with remarkable selectivity in organic and aqueous media. *J. Org. Chem.* **2011**, *76* (10), 3820-8.

26. Hu, R.; Feng, J.; Hu, D.; Wang, S.; Li, S.; Li, Y.; Yang, G. A rapid aqueous fluoride ion sensor with dual output modes. *Angew. Chem., Int. Ed.* **2010**, *49* (29), 4915-8.

Table S2. Cartesian Coordinates of the Optimized Geometries**:**

1

C	1.946222000	0.223813000	0.000123000
C	2.346435000	-1.126822000	0.000206000
C	2.933432000	1.226494000	-0.000125000
C	3.697247000	-1.458804000	0.000061000
C	4.287510000	0.891150000	-0.000261000
C	4.672446000	-0.451930000	-0.000170000
H	1.586431000	-1.900508000	0.000384000
H	2.632853000	2.271133000	-0.000200000
H	3.996952000	-2.502675000	0.000126000
H	5.039197000	1.674595000	-0.000444000
C	0.541537000	0.626054000	0.000276000
H	0.353348000	1.706968000	0.000147000
N	-0.433525000	-0.220864000	0.000171000
C	-1.756554000	0.203718000	0.000079000
C	-2.734500000	-0.772681000	-0.000041000
C	-2.103874000	1.591621000	0.000102000
N	-2.278899000	2.744247000	0.000082000
C	-4.126932000	-0.430343000	-0.000148000
N	-5.268859000	-0.208760000	-0.000235000
N	-2.437377000	-2.100396000	-0.000047000
H	-1.463246000	-2.365023000	0.000015000
H	-3.158538000	-2.801498000	-0.000155000
H	5.725806000	-0.716191000	-0.000284000

1+F⁻

C	2.019544000	0.502611000	-0.000061000
C	2.367719000	-0.864513000	-0.000675000
C	3.047993000	1.464003000	0.000178000
C	3.706919000	-1.248236000	-0.001042000
C	4.389458000	1.075033000	-0.000239000
C	4.724374000	-0.282678000	-0.000835000
H	1.575747000	-1.607151000	-0.000779000
H	2.790345000	2.520402000	0.000666000
H	3.963242000	-2.304113000	-0.001476000
H	5.170815000	1.829763000	-0.000096000
C	0.623830000	0.948809000	0.000446000
H	0.465754000	2.035783000	0.001440000
N	-0.361219000	0.114564000	-0.000295000
C	-1.684640000	0.506549000	0.000009000
C	-2.692443000	-0.484033000	-0.001091000
C	-2.042292000	1.883901000	0.001171000
N	-2.232790000	3.039895000	0.002042000
C	-4.065396000	-0.002875000	-0.001109000
N	-5.184525000	0.316835000	-0.001167000
N	-2.507194000	-1.797095000	-0.002292000
H	-1.383103000	-2.563545000	0.000993000
H	-3.377692000	-2.319885000	-0.003138000
H	5.766750000	-0.588199000	-0.001133000
F	-0.593573000	-3.301543000	0.003887000

2

C	-3.031176000	0.032097000	0.000111000
C	-2.153093000	-1.057271000	0.011973000
C	-2.561590000	1.345066000	-0.014576000
C	-0.782949000	-0.822333000	0.009165000
C	-1.185704000	1.568898000	-0.017443000
C	-0.280042000	0.493389000	-0.005666000
H	-2.536563000	-2.071235000	0.023351000
H	-3.254022000	2.179002000	-0.023627000
H	-0.090600000	-1.656947000	0.018476000

H	-0.812259000	2.588926000	-0.028914000
C	1.151977000	0.781111000	-0.009294000
H	1.426496000	1.842646000	-0.021051000
N	2.048963000	-0.147865000	0.000500000
C	3.404066000	0.148399000	0.000962000
C	4.291166000	-0.916724000	-0.006763000
C	3.889566000	1.492416000	0.006749000
N	4.201078000	2.616916000	0.013888000
C	5.706334000	-0.672983000	0.005924000
N	6.856225000	-0.499960000	0.020359000
N	3.891731000	-2.208879000	-0.049419000
H	2.900871000	-2.399484000	0.014641000
H	4.546304000	-2.964797000	0.078899000
Br	-4.910215000	-0.291200000	0.004402000

2+F⁻

C	-2.982393000	0.004936000	0.016419000
C	-2.040713000	-1.027121000	0.020497000
C	-2.591464000	1.340789000	-0.014466000
C	-0.682084000	-0.718422000	-0.006442000
C	-1.225012000	1.643947000	-0.036485000
C	-0.254874000	0.622400000	-0.031277000
H	-2.363149000	-2.062288000	0.045361000
H	-3.333219000	2.131889000	-0.017947000
H	0.055353000	-1.524489000	-0.003595000
H	-0.912932000	2.684672000	-0.053090000
C	1.177670000	0.954485000	-0.036201000
H	1.440996000	2.019564000	-0.032616000
N	2.070339000	0.022878000	-0.028294000
C	3.433195000	0.234718000	-0.000979000
C	4.249801000	-0.906684000	0.005240000
C	3.986629000	1.549339000	0.023797000
N	4.331577000	2.667748000	0.046001000
C	5.684484000	-0.725783000	0.040152000
N	6.843267000	-0.620034000	0.070517000
N	3.796170000	-2.165118000	-0.015010000
H	2.732361000	-2.480975000	-0.034774000
H	4.491920000	-2.900594000	-0.006568000
Br	-4.847792000	-0.427727000	0.046639000
F	1.466696000	-3.067180000	0.001854000

3

C	-1.208517000	3.482550000	0.083238000
C	-1.219074000	2.073145000	0.063994000
C	-0.004642000	4.182295000	0.016726000
C	-0.001828000	1.380352000	0.004351000
C	1.200469000	3.485182000	-0.055091000
C	1.214236000	2.075641000	-0.048295000
H	-0.005540000	5.267611000	0.021446000
H	-0.000946000	0.297199000	0.000369000
H	2.140223000	4.028669000	-0.105017000
C	-2.510053000	1.384435000	0.107765000
H	-3.386008000	2.019407000	0.288862000
N	-2.630619000	0.111050000	-0.062852000
C	-3.874979000	-0.506985000	-0.007715000
C	-3.920151000	-1.864106000	-0.256845000
C	-5.066103000	0.227707000	0.291877000
N	-5.971967000	0.917956000	0.539882000
C	-5.160712000	-2.582330000	-0.230035000
N	-6.139704000	-3.210481000	-0.230495000
N	-2.796329000	-2.600745000	-0.506809000
H	-1.953041000	-2.071256000	-0.683072000
H	-2.887108000	-3.508857000	-0.935422000
C	2.507191000	1.390284000	-0.093363000
H	3.382780000	2.029754000	-0.259437000
N	2.630183000	0.115179000	0.061466000
C	3.875062000	-0.500398000	-0.005296000
C	3.931410000	-1.848949000	0.289777000
C	5.055585000	0.227514000	-0.355991000
N	5.952452000	0.910623000	-0.652426000
C	5.165768000	-2.574704000	0.211278000
N	6.136925000	-3.211997000	0.151882000
N	2.839500000	-2.552774000	0.698540000
H	2.861326000	-3.558434000	0.743093000
H	1.952505000	-2.069315000	0.697613000
H	-2.149252000	4.023752000	0.139029000

3+F⁻

C	1.132616000	2.252267000	-0.002728000
C	-0.073398000	1.520299000	-0.000079000
C	1.071864000	3.658783000	-0.004633000
C	-1.298037000	2.184345000	0.000630000
C	-0.158067000	4.320671000	-0.003867000
C	-1.348025000	3.586135000	-0.001247000
H	-0.028228000	0.435546000	0.001312000
H	1.995639000	4.232366000	-0.006696000
H	-0.187191000	5.406620000	-0.005313000
C	2.441350000	1.593233000	-0.003662000
H	3.315466000	2.258376000	-0.006296000
N	2.560175000	0.307868000	-0.001458000
C	3.779338000	-0.339230000	-0.002120000
C	3.804911000	-1.752120000	0.000688000
C	4.999280000	0.393401000	-0.005331000
N	5.944877000	1.085102000	-0.007942000
C	5.121884000	-2.370259000	0.000220000
N	6.144609000	-2.925864000	-0.000006000
N	2.753054000	-2.559609000	0.003871000
H	1.517003000	-2.066326000	0.000714000
H	3.008250000	-3.542435000	0.005681000
H	-2.306027000	4.098113000	-0.000673000
F	0.436578000	-2.040130000	-0.001192000
C	-2.605480000	1.370597000	0.003477000
H	-3.549338000	1.874613000	0.004018000
C	-3.847878000	-0.421533000	0.007547000
N	-2.562753000	0.081306000	0.005208000
C	-3.801176000	-1.830758000	0.009440000
N	-2.637186000	-2.452504000	0.040405000
H	-1.790948000	-1.925768000	0.120591000
H	-2.601614000	-3.450260000	-0.016323000
C	-5.033821000	0.359453000	0.007981000
C	-5.006314000	-2.581129000	-0.021965000
N	-5.991428000	0.990072000	0.008332000
N	-5.979420000	-3.187028000	-0.047324000

4

C	-1.412215000	-0.879783000	-0.016556000
C	-0.691841000	0.335546000	0.001456000
C	-0.695568000	-2.090088000	-0.034669000
C	0.691839000	0.335545000	0.001519000
C	0.695567000	-2.090088000	-0.034582000
C	1.412213000	-0.879783000	-0.016421000
H	-1.241978000	1.269864000	0.014533000
H	-1.235985000	-3.032659000	-0.048955000
H	1.241979000	1.269861000	0.014652000
H	1.235984000	-3.032661000	-0.048794000
C	-2.869990000	-0.927335000	-0.017982000
H	-3.318417000	-1.928042000	-0.035517000
N	-3.605113000	0.135840000	-0.000130000
C	-4.990236000	0.051775000	0.005093000
C	-5.698369000	1.239278000	-0.005262000
C	-5.668324000	-1.208592000	0.018420000
N	-6.120675000	-2.282749000	0.031879000
C	-7.131904000	1.247490000	0.022966000
N	-8.292944000	1.310208000	0.055204000
N	-5.085796000	2.454418000	-0.074507000
H	-4.078998000	2.461436000	0.013083000
H	-5.601753000	3.295693000	0.127943000
C	2.869989000	-0.927341000	-0.017718000
H	3.318415000	-1.928057000	-0.034804000
N	3.605115000	0.135838000	-0.000215000
C	4.990236000	0.051770000	0.005187000
C	5.698367000	1.239277000	-0.005668000
C	5.668324000	-1.208584000	0.019155000
N	6.120674000	-2.282735000	0.033169000
C	7.131903000	1.247492000	0.022668000
N	8.292941000	1.310213000	0.054968000
N	5.085804000	2.454369000	-0.075490000
H	5.601778000	3.295800000	0.126249000
H	4.078986000	2.461472000	0.011842000

4 + F⁻

C	-1.578909000	-1.067791000	0.018364000
C	-0.811985000	0.121777000	0.015093000

C	-0.903633000	-2.304453000	0.023326000
C	0.571077000	0.074817000	0.015578000
C	0.486210000	-2.354267000	0.024701000
C	1.249290000	-1.169553000	0.019701000
H	-1.324808000	1.077891000	0.010780000
H	-1.477209000	-3.227649000	0.025929000
H	1.156323000	0.989645000	0.011592000
H	0.991742000	-3.316202000	0.028342000
C	-3.031924000	-1.061893000	0.013745000
H	-3.514832000	-2.046906000	0.007215000
N	-3.730988000	0.029171000	0.015315000
C	-5.115607000	0.007623000	-0.004377000
C	-5.775058000	1.226013000	0.035721000
C	-5.861195000	-1.211582000	-0.062243000
N	-6.395352000	-2.247841000	-0.112948000
C	-7.207896000	1.273460000	-0.008559000
N	-8.368980000	1.338449000	-0.050206000
N	-5.119612000	2.410294000	0.154350000
H	-4.111722000	2.385023000	0.068111000
H	-5.593687000	3.279696000	-0.037543000
C	2.705489000	-1.252536000	0.017233000
H	3.138869000	-2.260969000	0.025601000
N	3.440076000	-0.186970000	0.004388000
C	4.811832000	-0.206305000	0.001164000
C	5.505327000	1.030800000	-0.017499000
C	5.535774000	-1.432266000	0.015412000
N	6.039712000	-2.489302000	0.028048000
C	6.958610000	0.958321000	-0.017394000
N	8.122210000	0.965851000	-0.018166000
N	4.946988000	2.228458000	-0.035052000
H	3.607154000	2.614079000	-0.044227000
H	5.626394000	2.983536000	-0.046226000
F	2.637664000	3.043566000	-0.054385000

4 + OAc⁻

C	1.977684000	-1.518920000	-0.003888000
C	1.126093000	-0.389854000	-0.002623000
C	1.400422000	-2.802894000	-0.005395000
C	-0.250491000	-0.539308000	-0.003017000
C	0.017305000	-2.956553000	-0.005981000
C	-0.828376000	-1.831607000	-0.004745000
H	1.566348000	0.601502000	-0.000987000
H	2.041688000	-3.680114000	-0.006309000
H	-0.898526000	0.332524000	-0.001682000
H	-0.415122000	-3.953343000	-0.007260000
C	3.429542000	-1.402228000	-0.003394000
H	3.987339000	-2.346617000	-0.001047000
N	4.037831000	-0.260275000	-0.005411000
C	5.419992000	-0.167811000	0.002012000
C	5.973843000	1.103126000	-0.026045000
C	6.263635000	-1.321471000	0.034621000
N	6.880691000	-2.311487000	0.064209000
C	7.399408000	1.268975000	-0.003959000
N	8.551507000	1.428197000	0.018098000
N	5.223993000	2.228927000	-0.103927000
H	4.220346000	2.127577000	-0.029845000
H	5.633473000	3.138327000	0.043896000
C	-2.276032000	-2.027206000	-0.005015000
H	-2.630328000	-3.065752000	-0.006763000
N	-3.089720000	-1.024780000	-0.003233000
C	-4.461484000	-1.163548000	-0.003489000
C	-5.229299000	0.005587000	-0.000354000
C	-5.091218000	-2.443851000	-0.006704000
N	-5.514995000	-3.533003000	-0.009437000
C	-6.667472000	-0.115387000	-0.001035000
N	-7.828264000	-0.184791000	-0.001522000
N	-4.727431000	1.243228000	0.003257000
H	-3.705962000	1.478295000	0.005385000
H	-5.328219000	2.057087000	0.006104000
C	-2.659079000	3.681494000	0.015719000
O	-2.281559000	2.462075000	0.011836000
O	-3.860698000	4.070910000	0.015894000
C	-1.567247000	4.758672000	0.020001000
H	-1.678998000	5.398345000	-0.862403000
H	-1.686965000	5.400277000	0.899885000

H	-0.565663000	4.322601000	0.024688000

4 + CN^{-}

C	1.580773000	-1.177574000	0.013823000
C	0.826028000	0.017988000	0.010642000
C	0.899851000	-2.409715000	0.017810000
C	-0.558059000	-0.019127000	0.010568000
C	-0.491220000	-2.449061000	0.018274000
C	-1.240732000	-1.257697000	0.013954000
H	1.344806000	0.970450000	0.006979000
H	1.466947000	-3.336524000	0.020321000
H	-1.131037000	0.902751000	0.006846000
H	-1.004439000	-3.406710000	0.021141000
C	3.038226000	-1.180735000	0.010420000
H	3.516949000	-2.167546000	0.005709000
N	3.736453000	-0.092000000	0.011348000
C	5.121291000	-0.109824000	-0.002473000
C	5.774290000	1.113903000	0.023019000
C	5.870124000	-1.326675000	-0.040180000
N	6.405956000	-2.362880000	-0.073032000
C	7.208807000	1.164237000	-0.008961000
N	8.369815000	1.228612000	-0.038637000
N	5.117736000	2.294676000	0.100842000
H	4.107787000	2.276932000	0.049530000
H	5.599168000	3.172368000	-0.018114000
C	-2.698756000	-1.336900000	0.011398000
H	-3.128221000	-2.346547000	0.015360000
N	-3.441890000	-0.279942000	0.003938000
C	-4.821602000	-0.348873000	0.000207000
C	-5.545238000	0.842547000	-0.010230000
C	-5.511986000	-1.600223000	0.005647000
N	-5.987759000	-2.666959000	0.010688000
C	-6.985773000	0.768324000	-0.014425000
N	-8.148492000	0.743330000	-0.017912000
N	-5.013163000	2.074972000	-0.017520000
H	-3.991866000	2.285522000	-0.015284000
H	-5.629333000	2.875016000	-0.022753000
C	-2.320740000	3.354293000	-0.020296000
N	-1.327579000	3.989472000	-0.023275000

4 + Br^{-}

C	-1.887832000	-1.490526000	0.014285000
C	-1.081934000	-0.330770000	0.009008000
C	-1.262079000	-2.750493000	0.020042000
C	0.298124000	-0.424232000	0.009176000
C	0.123676000	-2.848974000	0.020276000
C	0.924016000	-1.691740000	0.014390000
H	-1.560440000	0.642605000	0.003889000
H	-1.870049000	-3.650905000	0.024235000
H	0.903029000	0.479512000	0.004516000
H	0.596440000	-3.827174000	0.024488000
C	-3.342526000	-1.426006000	0.012548000
H	-3.867479000	-2.389069000	0.010982000
N	-3.986534000	-0.305477000	0.011590000
C	-5.370841000	-0.259394000	-0.002876000
C	-5.962194000	0.994030000	0.032898000
C	-6.179351000	-1.436964000	-0.049853000
N	-6.768075000	-2.442869000	-0.091747000
C	-7.391311000	1.115711000	-0.007285000
N	-8.547454000	1.234548000	-0.045691000
N	-5.245131000	2.137397000	0.141292000
H	-4.241104000	2.049168000	0.057031000
H	-5.669405000	3.029059000	-0.059994000
C	2.375596000	-1.837918000	0.012654000
H	2.757736000	-2.866485000	0.018270000
N	3.166818000	-0.817806000	0.004183000
C	4.541664000	-0.952734000	0.001410000
C	5.310806000	0.207995000	-0.008955000
C	5.180453000	-2.231003000	0.008203000
N	5.615262000	-3.314083000	0.014240000
C	6.745317000	0.084170000	-0.012569000
N	7.905748000	0.012015000	-0.015710000
N	4.813755000	1.456764000	-0.016189000
H	3.816691000	1.629893000	-0.012405000
H	5.462245000	2.240877000	-0.023852000
Br	1.910345000	3.103205000	-0.014316000

4 + I^-

C	2.056982000	-1.876884000	0.000016000
C	1.211924000	-0.732146000	0.000060000
C	1.464643000	-3.165118000	-0.000043000
C	-0.176013000	-0.871401000	0.000049000
C	0.070702000	-3.306902000	-0.000064000
C	-0.769836000	-2.164846000	-0.000017000
H	1.664085000	0.254526000	0.000102000
H	2.098995000	-4.048879000	-0.000079000
H	-0.813942000	0.007844000	0.000078000
H	-0.372101000	-4.300517000	-0.000114000
C	3.516071000	-1.764792000	0.000027000
H	4.073127000	-2.710745000	0.000031000
N	4.126102000	-0.607488000	0.000028000
C	5.518544000	-0.468892000	0.000050000
C	6.011671000	0.839409000	-0.000041000
C	6.413618000	-1.587945000	0.000099000
N	7.095417000	-2.558649000	0.000117000
C	7.431166000	1.086100000	-0.000037000
N	8.593983000	1.296188000	-0.000014000
N	5.180350000	1.919896000	-0.000175000
H	4.181430000	1.743077000	-0.000164000
H	5.526666000	2.869113000	-0.000079000
C	-2.222577000	-2.349798000	-0.000039000
H	-2.576810000	-3.389057000	-0.000081000
N	-3.052064000	-1.338456000	0.000015000
C	-4.441951000	-1.483331000	0.000010000
C	-5.203339000	-0.307913000	0.000055000
C	-5.086304000	-2.763976000	-0.000070000
N	-5.547878000	-3.857158000	-0.000120000
C	-6.644487000	-0.391796000	0.000029000
N	-7.824672000	-0.449004000	0.000029000
N	-4.648756000	0.934631000	0.000107000
H	-3.641555000	1.032926000	0.000111000
H	-5.237011000	1.764920000	0.000141000
I	-1.646512000	3.155820000	-0.000007000

4 + PO_4^-

C	-2.476961000	-1.668231000	0.016623000
C	-1.507519000	-0.632255000	0.012797000
C	-2.020628000	-3.005618000	0.021060000
C	-0.157182000	-0.914586000	0.012886000
C	-0.666236000	-3.293833000	0.022441000
C	0.298635000	-2.261127000	0.017441000
H	-1.847479000	0.398086000	0.008592000
H	-2.747017000	-3.814740000	0.023905000
H	0.568799000	-0.105838000	0.009592000
H	-0.333673000	-4.328832000	0.026362000
C	-3.894157000	-1.405059000	0.014489000
H	-4.547500000	-2.286692000	0.006655000
N	-4.392399000	-0.200607000	0.020630000
C	-5.750698000	0.025578000	-0.004238000
C	-6.190522000	1.342458000	0.059020000
C	-6.705640000	-1.038106000	-0.088671000
N	-7.422586000	-1.955393000	-0.164099000
C	-7.582596000	1.646734000	-0.002368000
N	-8.715431000	1.917148000	-0.062690000
N	-5.315986000	2.384662000	0.243250000
H	-4.345532000	2.126381000	0.105177000
H	-5.581741000	3.291564000	-0.113568000
C	1.707708000	-2.595589000	0.016952000
H	1.965862000	-3.662504000	0.030476000
N	2.615327000	-1.667431000	0.000881000
C	3.964228000	-1.880866000	0.002068000
C	4.776933000	-0.715163000	-0.020598000
C	4.536989000	-3.181821000	0.025172000
N	4.927866000	-4.284794000	0.044650000
C	6.209787000	-0.872843000	-0.015768000
N	7.368084000	-0.989838000	-0.013698000
N	4.273265000	0.508906000	-0.044648000
H	3.262950000	0.566958000	-0.040671000
H	4.827832000	1.361794000	-0.066320000
P	3.148201000	3.397338000	-0.014114000
O	2.784573000	4.251852000	-1.268331000
O	2.245206000	2.089652000	-0.002021000

O	2.901618000	4.198757000	1.301393000
O	4.685435000	2.985634000	-0.091571000

4 + HSO4⁻

C	-3.082751000	-1.453656000	0.032072000
C	-2.030844000	-0.512158000	-0.016860000
C	-2.764766000	-2.823112000	0.074708000
C	-0.712751000	-0.928826000	-0.022189000
C	-1.439991000	-3.241785000	0.068141000
C	-0.392645000	-2.304206000	0.020033000
H	-2.273278000	0.544308000	-0.050136000
H	-3.564662000	-3.557248000	0.112671000
H	0.093542000	-0.204726000	-0.059438000
H	-1.207182000	-4.302334000	0.100815000
C	-4.483054000	-1.054181000	0.039634000
H	-5.215052000	-1.869624000	0.082777000
N	-4.851681000	0.183801000	-0.002288000
C	-6.189264000	0.544702000	-0.003178000
C	-6.480581000	1.900033000	-0.023384000
C	-7.244614000	-0.418788000	0.018759000
N	-8.046424000	-1.265494000	0.034620000
C	-7.846223000	2.339765000	-0.049681000
N	-8.946475000	2.714958000	-0.077472000
N	-5.522234000	2.855157000	0.015320000
H	-4.565934000	2.539361000	-0.075823000
H	-5.739714000	3.810937000	-0.218918000
C	0.986107000	-2.780119000	0.015028000
H	1.121614000	-3.867909000	0.044369000
N	1.991810000	-1.970751000	-0.021336000
C	3.299694000	-2.419393000	-0.026176000
C	4.303263000	-1.449980000	-0.063790000
C	3.631353000	-3.805899000	0.006610000
N	3.815684000	-4.958273000	0.035529000
C	5.681256000	-1.868432000	-0.067148000
N	6.795381000	-2.199164000	-0.069507000
N	4.064496000	-0.135576000	-0.100043000
H	3.094927000	0.153808000	-0.082252000
H	4.801048000	0.566481000	-0.093779000
O	5.137023000	4.288190000	-1.072046000
O	3.388853000	2.814404000	-0.053416000
O	5.769031000	2.097461000	-0.094538000
O	4.982734000	3.946618000	1.408692000
H	5.889883000	4.291382000	1.452817000
S	4.802165000	3.237862000	-0.090118000

4 + Cl⁻

C	-1.661807000	-1.231679000	0.020404000
C	-0.892502000	-0.047110000	0.013342000
C	-0.997623000	-2.471779000	0.029420000
C	0.489673000	-0.099006000	0.014325000
C	0.390550000	-2.527940000	0.030937000
C	1.154240000	-1.346573000	0.022534000
H	-1.402158000	0.910218000	0.005569000
H	-1.577847000	-3.390296000	0.034954000
H	1.072556000	0.818559000	0.007658000
H	0.893431000	-3.490956000	0.037523000
C	-3.117785000	-1.212512000	0.015988000
H	-3.612137000	-2.191616000	0.012492000
N	-3.796916000	-0.112891000	0.014348000
C	-5.181967000	-0.111383000	-0.005385000
C	-5.814103000	1.121851000	0.033051000
C	-5.951656000	-1.314356000	-0.060930000
N	-6.507301000	-2.338593000	-0.109723000
C	-7.246186000	1.196513000	-0.015244000
N	-8.405368000	1.277314000	-0.060388000
N	-5.135587000	2.287641000	0.151238000
H	-4.128771000	2.232814000	0.071803000
H	-5.587652000	3.165885000	-0.048755000
C	2.609906000	-1.443184000	0.019981000
H	3.028885000	-2.457254000	0.029123000
N	3.362100000	-0.394211000	0.005778000
C	4.740643000	-0.474478000	-0.000046000
C	5.462135000	0.716464000	-0.019493000
C	5.429683000	-1.726267000	0.011298000
N	5.907021000	-2.791266000	0.021504000
C	6.900619000	0.648974000	-0.028336000

N	8.062920000	0.621858000	-0.035913000
N	4.916686000	1.944529000	-0.031748000
H	3.914256000	2.082938000	-0.021782000
H	5.537470000	2.750659000	-0.046351000
Cl	2.067892000	3.414305000	-0.015234000

4 + NO3⁻

C	1.947205000	-1.478903000	0.010921000
C	1.119649000	-0.334313000	0.003762000
C	1.344674000	-2.749851000	0.018411000
C	-0.258547000	-0.453560000	0.004401000
C	-0.039004000	-2.873839000	0.018903000
C	-0.861911000	-1.732639000	0.012179000
H	1.582797000	0.646419000	-0.002974000
H	1.968949000	-3.639045000	0.023826000
H	-0.882745000	0.434705000	-0.001490000
H	-0.493351000	-3.860687000	0.024540000
C	3.400208000	-1.387087000	0.009865000
H	3.942722000	-2.340345000	0.010747000
N	4.023844000	-0.254958000	0.007298000
C	5.407275000	-0.184809000	-0.005133000
C	5.977520000	1.078312000	0.028442000
C	6.235832000	-1.348607000	-0.047525000
N	6.841548000	-2.344507000	-0.085734000
C	7.404464000	1.223533000	-0.008726000
N	8.558571000	1.361457000	-0.044707000
N	5.241518000	2.210264000	0.132622000
H	4.239467000	2.105234000	0.044633000
H	5.651517000	3.107615000	-0.073167000
C	-2.310654000	-1.909033000	0.011540000
H	-2.670809000	-2.945557000	0.011054000
N	-3.122402000	-0.905466000	0.010032000
C	-4.494406000	-1.063495000	0.002765000
C	-5.280272000	0.089131000	0.003031000
C	-5.111388000	-2.351280000	-0.007527000
N	-5.528347000	-3.441682000	-0.015560000
C	-6.713762000	-0.056294000	-0.014259000
N	-7.872616000	-0.147792000	-0.028504000
N	-4.797764000	1.339638000	0.016186000
H	-3.802238000	1.519041000	0.043855000
H	-5.451506000	2.119351000	0.011069000
N	-2.390042000	3.603193000	-0.010517000
O	-2.194755000	2.346627000	0.110050000
O	-3.567437000	4.040329000	-0.077855000
O	-1.408591000	4.376625000	-0.057970000

Table S3. Production cost of sensor **4**

Item	Sigma-Aldrich price
Terephthalaldehyde (USD/mmol)	0.0773 (500 g/288 USD)
DAMN (USD/mmol)	0.0989 (100 g/91.5 USD)
Methanol (USD/L)	40.336 (2.5 L/ 100.84 USD)
H_2SO_4 (USD/L)	120.48 (1L/ 120.48 USD)
Total cost 10 mmol scale (88% yield) (USD/g)	1.73
Total cost 100 mmol scale (96% yield) (USD/g)	1.59

-----------END---------
